# Eliminate pneumococcal colonization by targeting intracellular acidification that promotes H_2_O_2_ production to enhance bacterial survival

**DOI:** 10.1371/journal.ppat.1014381

**Published:** 2026-06-23

**Authors:** Chengwang Zhang, Qingxiu Gan, Yu He, Yuting Zhang, Xuan Wu, Shimi Lv

**Affiliations:** Department of Basic Medical Science, School of Medicine, Lishui University, Lishui, Zhejiang, China; INEM: Institut Necker-Enfants Malades, FRANCE

## Abstract

*Streptococcus pneumoniae* is an important human pathogen that causes severe threat to the lives of children under 5 years old and the elder. Colonization in the nasopharynx is the prerequisite for pneumococcal disease. However, few attentions have been paid to prevent pneumococcal disease by eliminating colonization. In this work, pneumococci were found to undergo methionine starvation in the nasopharynx. In the *in vitro* experiments, methionine starvation induces the cytoplasmic acidification of *S. pneumoniae*, which benefits bacterial survival. Intracellular acidification was also observed in pneumococci colonizing the nasopharynx. We found that increased intracellular lactate level under methionine starvation causes intracellular acidification. Surprisingly, intracellular acidification elevates intracellular level of H_2_O_2_, a metabolite commonly considered harmful for bacteria, to enhance bacterial survival under methionine starvation. H_2_O_2_ inhibits bacterial autolysis that can be induced by methionine starvation to enhance bacterial survival. To impair pneumococcal survival and colonization, sodium oxamate was used as a drug by elevating intracellular pH through inhibiting lactate production. Interestingly, we found that penicillin alone could not impair pneumococcal survival and colonization efficiently due to the inhibited killing by intracellular acidification. However, the combination of sodium oxamate and penicillin not only killed bacteria effectively, but also almost eradicated pneumococcal colonization. To the best of our knowledge, it is the first time that H_2_O_2_ production was reported to be induced by intracellular acidification to benefit bacterial survival. Besides, sodium oxamate was found to be a novel drug for eradicating pneumococcal colonization by targeting intracellular acidification, particularly in the combination with penicillin.

## Author summary

The essential role of nasal colonization for *S. pneumoniae* to cause diseases makes it urgent to know how this bacterium survives in the nasopharynx, an environment with poor nutrients. *S. pneumoniae* experiences methionine starvation during nasal colonization. This study finds that *S. pneumoniae* enhances its survival both in the *in vitro* culture and in the nasopharynx by intracellular acidification realized by increased intracellular lactate level under methionine starvation. Interestingly, intracellular acidification promotes H_2_O_2_ production that inhibits bacterial autolysis under methionine starvation to enhance bacterial survival. Sodium oxamate, the inhibitor of lactate production, was used to broke intracellular acidification under methionine starvation to attenuate bacterial survival and colonization. The combination use of sodium oxamate and penicillin almost eliminates pneumococcal colonization. This study reveals how *S. pneumoniae* utilizes methionine starvation that causes intracellular acidification to enhance its survival in the nasopharynx. Sodium oxamate becomes a potential drug to eliminate pneumococcal colonization by targeting intracellular acidification. This effective way of eliminating colonization may eventually become a strategy to prevent pneumococcal disease.

## Introduction

*Streptococcus pneumoniae* is an important human pathogen. Pneumococcal disease is the main cause of the death of children under 5 years old [1]. In 2015, *S. pneumoniae* caused the death of 294,000 children under 5 years old (HIV-uninfected) globally [2]. In 2017, *S. pneumoniae* caused 218,000 severe pneumococcal cases (pneumoniae, meningitis, and etc.) and 8,000 deaths of children under 5 years old in China [3]. Although many antibiotics are used to treat pneumococcal disease, *S. pneumoniae* develops a large range of antibiotic resistance, such as penicillin, erythromycin, clindamycin, and tetracycline [4]. Pneumococcal conjugate vaccine (PCV) solved the problem of antibiotic resistance to some degree [5]. However, the serotypes not covered by PCV have exhibited antibiotic resistance [6]. Therefore, it is urgent to develop novel drugs for treating or preventing pneumococcal disease.

Pneumococcal colonization is the prerequisite for the invasive disease elicited by it [7]. We believed that eliminating pneumococcal colonization is an effective way to prevent pneumococcal disease. Bacteriophage lytic enzymes called lysins had been used to kill bacteria at human mucous membranes [8]. Lysins kill bacteria by digesting the cell wall^8^. The work by Loeffler et al. showed that 5 hours after pneumococcal lysin (Pal, 1,400 U) treatment, *S. pneumoniae* serotype 14 in the nasopharynx cannot be detected [9]. The use of anti-attachment substance, for example *S*-carboxymethylcysteine (*S*-CMC) is another strategy for eliminating pneumococcal colonization [10]. *S*-CMC is a mucolytic substance that can inhibit pneumococcal attachment to epithelial cells [10]. Besides, *S*-CMC promotes the detachment of *S. pneumoniae* from epithelial cells [10]. However, *S*-CMC did not kill bacteria [10]. Whether *S*-CMC can eliminate pneumococcal colonization is not clear.

What is easily to be ignored is the harsh environment of nasopharynx. Different from blood, the nutrition contents are relatively poor in the nasopharynx [11]. Glucose is 4–8 mM is the plasma and 0.04-1 mM in the nasal secretions [11]. Amino acids are 2.6-4.3 mM in the plasma and 0.65-2.2 mM in the nasal secretions [11]. The doubling time of *S. pneumoniae* is 40 min in THY medium, 108 min in the lung, and 161 min in the nasopharynx of mice [12]. This obviously longer doubling time indicates nutrition shortage for *S. pneumoniae* in the nasopharynx. Targeting the pathway of pneumococcal adaptation to nutrition starvation in the nasopharynx is an effective way to eradicate pneumococcal colonization.

Methionine is an important amino acid. It is not only used for protein translation either by direct usage or as the precursor of *N*-formylmethionine, the first used substance for all protein translation in bacteria [13], but also used as the precursor of *S*-adenosylmethionine, an important methyl donor [14]. Our previous work shows that the deletion of methionine synthesis gene *metE* attenuated pneumococcal colonization in the nasopharynx of mice significantly [15]. Besides, pneumococci collected from the nasopharynx of mice exhibited highly activated transcription of *metE* [15]. The work by Krismer et al. showed that methionine was not detected in the nasal secretions of human and the content of cysteine, the precursor for methionine synthesis, is extremely low [11]. These data indicate the shortage of methionine for pneumococcal survival in the nasopharynx.

Our previous work created a methionine starvation state by culturing *metE* deletion strain of pneumococcal D39 strain in chemically defined medium (CDM) with low concentration of methionine (1 μg/ml) [15,16]. Bacteria usually maintains a homeostasis of their intracellular pH, due to its important roles in controlling enzyme activity, redox potential, nucleic acid structure, and etc. [17–19]. Our previous work showed that *S. pneumoniae* maintains an intracellular pH ~ 7.6 normally [16]. Interestingly, methionine starvation induces cytoplasmic acidification of pneumococci [16]. This intracellular acidification is essential for enhanced bacterial survival under methionine starvation. The carriage duration of *S. pneumoniae* can be 13–65 days for infants, which shows a strong survival ability of *S. pneumoniae* in the nasopharynx [20]. Based on the studies above, we hypothesized that intracellular acidification enhances pneumococcal colonization in the nasopharynx. Inhibiting intracellular acidification may be a novel strategy to eradicate pneumococcal colonization.

However, there are still some key questions not answered. Firstly, how was bacterial cytoplasm acidified? Secondly, how does intracellular acidification enhance bacterial survival (which biological process was influenced)? *S. pneumoniae* is characterized by secreting high amounts of H_2_O_2_ [21]. SpxB is an enzyme of *S. pneumoniae* that can produce H_2_O_2_. Deletion of *spxB* gene showed weakness in the competitive colonization model with the strain having *spxB* gene [22]. Besides, The produced H_2_O_2_ causes apoptosis and DNA damage and in lung cells [23]. Therefore, it is worthy to study whether intracellular acidification regulates H_2_O_2_ production to influence pneumococcal survival. Thirdly, whether the cytoplasm of pneumococci is acidified during its colonization. Fourthly, how to target intracellular acidification to impair pneumococcal survival and colonization? In this work, we systematically explained how intracellular acidification functions and how to target intracellular acidification. Based on the novel theories we found in this work, a novel drug and a novel therapy were found to eliminate pneumococcal colonization. We believed that the findings in this work are extremely promising for eradicating pneumococcal colonization in the clinical application to prevent pneumococcal disease in human.

## Results

### Intracellular acidification enhances pneumococcal survival under methionine starvation

**Fig 1:**
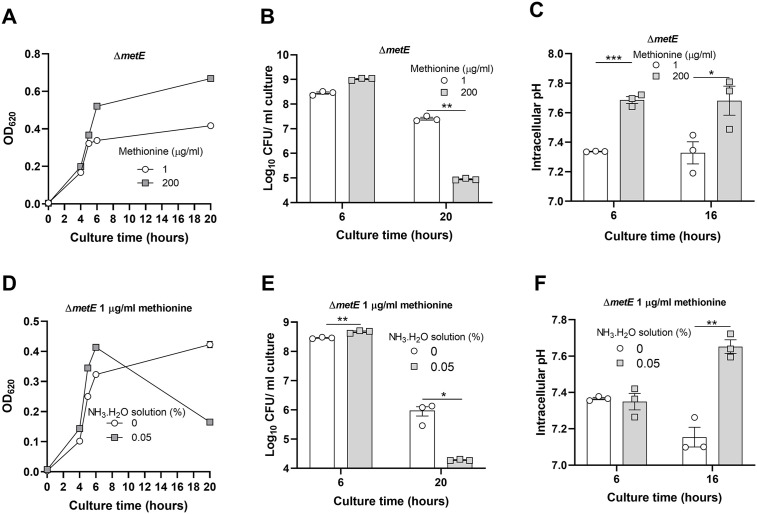
Enhanced survival by intracellular acidification. **A,** Growth curves (OD_620_), **B,** Survival (Colony forming unit (CFU)) and **C,** Intracellular pH of Δ*metE* cultured in CDM with 1 or 200 μg/ml methionine. At 6 and 20 hr post inoculation, bacterial CFU was determined. **D,** Growth curves (OD_620_), **e,** Survival (CFU) and **f,** Intracellular pH of Δ*metE* cultured in CDM with 1 μg/ml methionine and supplied with 0 or 0.05% NH_3_.H_2_O solution. At 6 and 20 hr post inoculation, bacterial CFU was determined. Each experiment was conducted in triplicate samples. P values < 0.05 (*), < 0.01 (**), and < 0.001 (***).

Deletion of methionine synthesis gene *metE* in the wild-type D39 strain (Δ*metE*) attenuated pneumococcal growth when limited methionine (1 μg/ml) was supplied (methionine starvation, Fig 1A). However, its long-term survival was enhanced under methionine starvation in the stationary phase. Although Δ*metE* cultured with 1 μg/ml methionine had a lower number of colony forming unit (CFU) at 6 hr post inoculation (Fig 1B), its CFU was significantly higher than the culture with 200 μg/ml methionine at 20 hr post inoculation (Fig 1B). Under this starvation, bacterial cytoplasm was acidified from pH 7.68 to 7.33 at 6 hr post inoculation (Fig 1C). At 16 hr post inoculation, this intracellular acidification still existed (Fig 1C).

To verify the enhancement of bacterial survival by intracellular acidification, bacterial culture was supplied with 0.05% NH_3_.H_2_O solution to determine its growth, survival and intracellular pH. Supply of NH_3_.H_2_O solution enhanced bacterial growth. Bacterial OD_620_ value increased from 0.34 (no supply) to 0.41 at 6 hr post inoculation by NH_3_.H_2_O supply (Fig 1D). Interestingly, bacteria then underwent autolysis (Fig 1D). At 20 hr post inoculation, the OD_620_ value of bacterial culture with NH_3_.H_2_O supply was only 0.16, compared to 0.42 with no NH_3_.H_2_O supply. At 6 hr post inoculation, bacterial CFU with NH_3_.H_2_O supply was 1.6-fold of the CFU without NH_3_.H_2_O supply. However, at 20 hr post inoculation, bacterial CFU with NH_3_.H_2_O supply was only 2% of the CFU without NH_3_.H_2_O supply (Fig 1E). Although intracellular pH was not elevated at 6 hr post inoculation, it increased to 7.65 at 16 hr post inoculation (Fig 1F). Enhanced survival under intracellular acidification and attenuated survival by elevating intracellular pH verified that it is intracellular acidification that enhances bacterial survival.

### Intracellular acidification was achieved by enhanced intracellular lactate production

**Fig 2:**
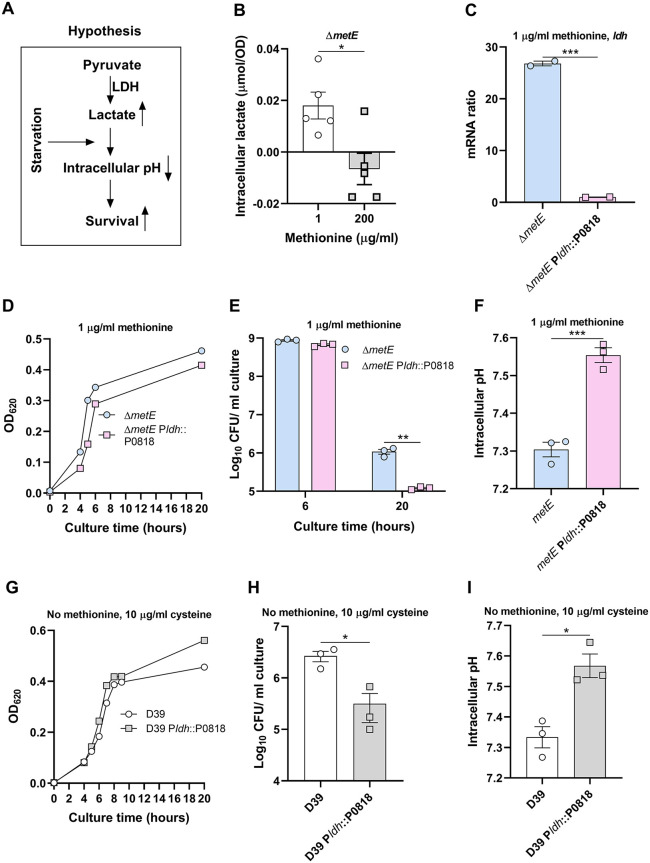
Enhanced lactate production under methionine starvation. **A,** Hypothesis of how intracellular acidification is realized. Under methionine starvation, lactate production from pyruvate by lactate dehydrogenase (LDH) increases, thus decreasing intracellular pH. Therefore, bacterial survival is enhanced. **B,** Intracellular lactate (μmol/OD bacteria) of Δ*metE* cultured in CDM with 1 or 200 μg/ml methionine. At 6 hr post inoculation, bacteria were collected to determine intracellular L-lactate. Each column has 5 samples. **C,** Comparison of *ldh* transcription level in Δ*metE* and Δ*metE* P*ldh*::P0818 (the promoter of *ldh* was replaced with the promoter of SPD0818 in Δ*metE* background). mRNA level of *ldh* in Δ*metE* P*ldh*::P0818 was set as 1. Each data point represents the mean value of three technical repeats. Experiment was repeated once. **D,** Growth curves (OD_620_), **E,** Survival (CFU) and **F,** Intracellular pH of Δ*metE* and Δ*metE* P*ldh*::P0818 cultured in CDM with 1 μg/ml methionine. At 6 and 20 hr post inoculation, bacterial CFU was determined. **G,** Growth curves (OD_620_), **H,** Survival (CFU, 20 hr post inoculation) and **I,** Intracellular pH (9 hr post inoculation) of D39 and D39 P*ldh*::P0818 (the promoter of *ldh* was replaced with the promoter of SPD0818 in D39 background) cultured in CDM with no methionine and 10 μg/ml cysteine. Each experiment was conducted in triplicate samples unless otherwise specified. P values < 0.05 (*), < 0.01 (**), and < 0.001 (***).

Lactate is an organic acid that is able to decrease intracellular pH [24]. Lactate is produced from pyruvate by lactate dehydrogenase (LDH). We hypothesized that intracellular lactate accumulation decreases intracellular pH to enhance bacterial survival (Fig 2A). To verify this hypothesis, intracellular lactate level was determined by a lactic acid content assay kit (Solarbio, Beijing, China). Methionine starvation did increase intracellular lactate level from not detected (Δ*metE*, 200 μg/ml methionine, the mean value below zero) to 0.018 μmol/OD (Δ*metE*, 1 μg/ml methionine) (Fig 2B). Therefore, we determined the importance of lactate synthesis gene *ldh* for intracellular acidification and enhanced survival under methionine starvation. The promoter of *ldh* was replaced by the promoter of SPD0818, a gene with lower reads, compared to the reads of *ldh* (~10%) in the RNA-seq result of methionine starvation (Δ*metE*, 1 μg/ml methionine) [15]. The q RT-PCR data showed that this promoter replacement down-regulated the transcription of *ldh* to 3.7% to the transcription of *ldh* with original promoter (Fig 2C). This promoter replacement impaired bacterial growth slightly (Fig 2D). At 6 hr and 20 hr post inoculation, the CFU of bacteria with original *ldh* promoter was 1.3-fold and 8.9-fold of the bacteria with replaced *ldh* promoter, which showed the significant attenuation of bacterial survival by this promoter replacement (Fig 2E). Besides, promoter replacement of *ldh* increased bacterial intracellular pH from 7.30 to 7.55 under methionine starvation (Fig 2F). Taken together, these data emphasized the importance of lactate production for intracellular acidification and enhanced bacterial survival under methionine starvation.

To exclude the possible effect caused by *metE* deletion, no methionine and reduced cysteine (the precursor for methionine synthesis) concentration (10 μg/ml) in CDM was used to culture wild-type strain (D39) to create a methionine starvation condition. Promoter replacement of *ldh* with the promoter of SPD0818 was also performed in D39 background. This promoter replacement did not impair bacterial growth (**Fig 2G**). However, bacterial survival was attenuated significantly (**Fig 2H**). Bacterial intracellular pH increased from 7.33 to 7.57 by this promoter replacement (**Fig 2I**). These data excluded the effect of gene deletion of *metE*.

### Intracellular H_2_O_2_ accumulation enhances pneumococcal survival under methionine starvation

**Fig 3:**
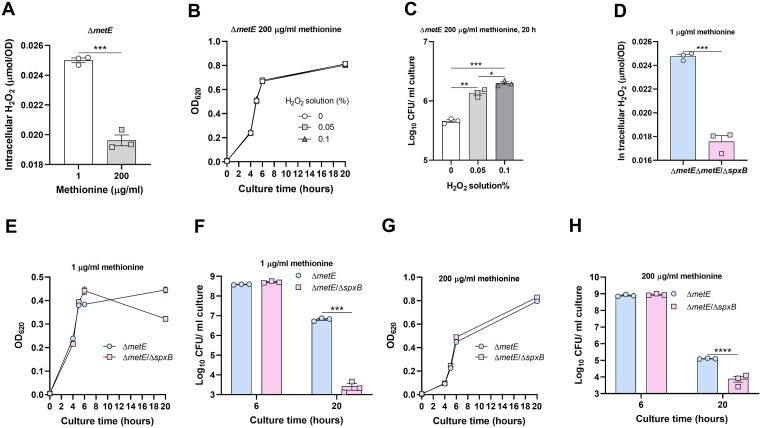
Enhanced survival by increased intracellular H_2_O_2_ level under methionine starvation. **A,** Intracellular H_2_O_2_ (μmol/OD bacteria) of Δ*metE* cultured in CDM with 1 or 200 μg/ml methionine. At 6 hr post inoculation, bacteria were collected to determine intracellular H_2_O_2_ level. **B,** Growth curves (OD_620_), **C,** Survival (CFU, 20 hr post inoculation) of Δ*metE* cultured in CDM with 200 μg/ml methionine and 0, 0.05% or 0.1% H_2_O_2_ solution. At 6 hr post inoculation, H_2_O_2_ solution was added. **D,** Intracellular H_2_O_2_ (μmol/OD bacteria) of Δ*metE* and Δ*metE*/Δ*spxB* cultured in CDM with 1 μg/ml methionine. At 6 hr post inoculation, bacteria were collected to determine intracellular H_2_O_2_ level. **E,** Growth curves (OD_620_) and **F,** Survival (CFU) of Δ*metE* and Δ*metE*/Δ*spxB* cultured in CDM with 1 μg/ml methionine. At 6 and 20 hr post inoculation, bacterial CFU was determined. **G,** Growth curves (OD_620_) and **H,** Survival (CFU) of Δ*metE* and Δ*metE*/Δ*spxB* cultured in CDM with 200 μg/ml methionine. At 6 and 20 hr post inoculation, bacterial CFU was determined. Each experiment was conducted in triplicate samples. P values < 0.05 (*), < 0.01 (**), < 0.001 (***), and < 0.0001 (****).

*S. pneumoniae* is characterized by producing hydrogen peroxide (H_2_O_2_) [21]. H_2_O_2_ is generally considered as a hazardous agent for bacteria. We hypothesized that intracellular H_2_O_2_ level is decreased under methionine starvation, so that bacterial survival is enhanced. To our great surprise, intracellular H_2_O_2_ of Δ*metE* cultured with 1 μg/ml methionine (0.0250 μmol/OD) is significantly higher than the culture with 200 μg/ml methionine (0.0196 μmol/OD) (Fig 3A). This result indicated that intracellular H_2_O_2_ accumulation contributes to enhanced bacterial survival under methionine starvation. To verify this, the culture of Δ*metE* with sufficient methionine (200 μg/ml) was supplied with different concentrations of H_2_O_2_ at 6 hr post inoculation. Bacterial growth was not altered by H_2_O_2_ addition (Fig 3B). At 6 hr post inoculation (before H_2_O_2_ addition), bacterial CFU was consistent (S1 Fig). At 20 hr post inoculation (14 hr post H_2_O_2_ addition), H_2_O_2_ addition significantly enhanced bacterial survival. The CFU of bacteria with addition of 0.05% or 0.1% H_2_O_2_ solution was 3.0- and 4.3-fold of bacterial culture without H_2_O_2_ addition respectively (Fig 3C). These data showed that H_2_O_2_ did enhance bacterial survival.

SpxB is the key enzyme responsible for H_2_O_2_ production in *S. pneumoniae* [21]. To further verify it is the intracellular H_2_O_2_ accumulation that contributes to enhanced bacterial survival under methionine starvation, *spxB* gene was deleted to determine its impact. Deletion of *spxB* in Δ*metE* background significantly reduced intracellular H_2_O_2_ level under methionine starvation (**Fig 3D**), which showed the importance of *spxB* for intracellular H_2_O_2_ level. Interestingly, deletion of s*pxB* caused bacterial autolysis under methionine starvation (**Fig 3E**). The OD_620_ value of Δ*metE*/Δ*spxB* decreased from 0.442 at 6 hr post inoculation to 0.322 at 20 hr post inoculation, which showed obvious autolysis. However, the culture of Δ*metE* did not show autolysis. At 6 hr post inoculation, deletion of *spxB* did not make difference to bacterial CFU, while at 20 hr post inoculation, the CFU of Δ*metE*/Δ*spxB* was only 0.041% of Δ*metE* (**Fig 3F**). To determine whether SpxB only enhances bacterial survival under methionine starvation, Δ*metE* and Δ*metE*/Δ*spxB* were cultured in CDM with sufficient methionine (200 μg/ml). Although bacterial growth was consistent (**Fig 3G**), the CFU of Δ*metE*/Δ*spxB* was 6.2% of Δ*metE* (**Fig 3H**), which shows the importance of SpxB in this non-starving condition. However, this percentage (6.2%) is extremely higher than 0.041% under methionine starvation. Take together, SpxB mainly contributes to bacterial survival under methionine starvation, which verified the importance of H_2_O_2_ in this condition.

LctO is also able to produce H_2_O_2_ [25]. To compare the importance of *lctO* and *spxB* for pneumococcal survival under methionine starvation, *lctO* was deleted in Δ*metE* background. In the culture with 1 μg/ml methionine, Δ*metE*/Δ*spxB* also showed obvious autolysis (**S2A Fig**). However, Δ*metE*/Δ*lctO* did not show autolysis (**S2B Fig**). At 8 hr post inoculation, there was no obvious difference between their CFUs (**S2B Fig**). However, at 20 hr post inoculation, the CFU of Δ*metE*/Δ*spxB* was only 0.11% of Δ*metE*/Δ*lctO* (**S2B Fig**). These results indicated that *spxB* is more important than *lctO* for pneumococcal survival under methionine starvation. Interestingly, double deletions of *spxB* and *lctO* in Δ*metE* background made bacterial fail to grow in the same culture (**S2A Fig**), which showed the importance of *lctO* for pneumococcal growth in this culture when *spxB* was absent.

### Intracellular acidification promotes H_2_O_2_ production

**Fig 4:**
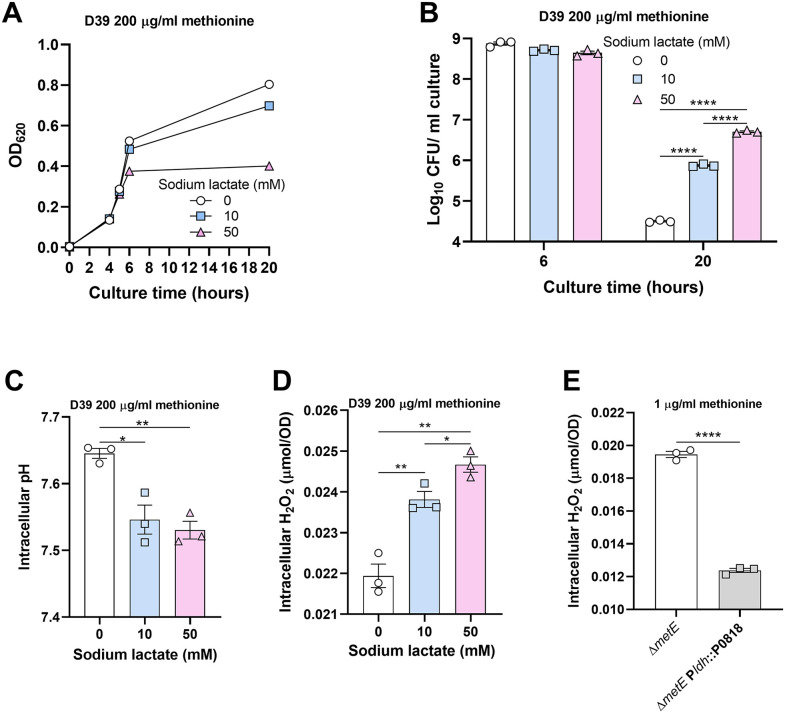
Increased intracellular H_2_O_2_ level by intracellular acidification. **A,** Growth curves (OD_620_), **B,** Survival (CFU), **C,** Intracellular pH and **D,** Intracellular H_2_O_2_ (μmol/OD bacteria) of wild-type strain (D39) cultured in CDM with 200 μg/ml methionine and 0-, 10-, or 50 mM sodium lactate. At 6 and 20 hr post inoculation, bacterial CFU was determined. **E,** Intracellular H_2_O_2_ (μmol/OD bacteria) of Δ*metE* and Δ*metE* P*ldh*::P0818 cultured in CDM with 1 μg/ml methionine. Each experiment was conducted in triplicate samples. P values < 0.05 (*), < 0.01 (**), and < 0.0001 (****).

Methionine starvation induces intracellular acidification as well as elevating intracellular H_2_O_2_ level. We therefore hypothesized that intracellular acidification promotes H_2_O_2_ production. To verify this, D39 was cultured in CDM supplemented with different concentrations of sodium lactate at 4 hr post inoculation to make different intracellular pH of bacteria. Addition of 10 mM sodium lactate reduced bacterial growth slightly and addition of 50 mM sodium lactate reduced bacterial growth severely (**Fig 4A**). At 6 hr post inoculation, bacterial CFU was almost consistent. However, at 20 hr post inoculation, the CFU of bacteria cultured with 10- or 50 mM sodium lactate was 23.7- and 160.5-fold of the culture without sodium lactate supply respectively (**Fig 4B**). Addition of 10- or 50 mM sodium lactate decreased bacterial intracellular pH from 7.64 to 7.55 and 7.53 respectively at 6 hr post inoculation (**Fig 4C**). These data again verified the survival enhancement by intracellular acidification. Interestingly, sodium lactate supply increased intracellular H_2_O_2_ level (**Fig 4D**), which shows the enhanced H_2_O_2_ production by intracellular acidification. To confirm this phenomenon, the intracellular H_2_O_2_ levels of Δ*metE* and Δ*metE* P***ldh***::P0818 (with a higher intracellular pH than Δ*metE* under methionine starvation) were compared. Δ*metE* P***ldh***::P0818 had a significantly lower intracellular H_2_O_2_ level than Δ*metE* (**Fig 4E**). Taken together, these data show that intracellular acidification promotes H_2_O_2_ production to enhance bacterial survival.

### H_2_O_2_ enhances bacterial survival under methionine starvation by inhibiting autolysis

**Fig 5:**
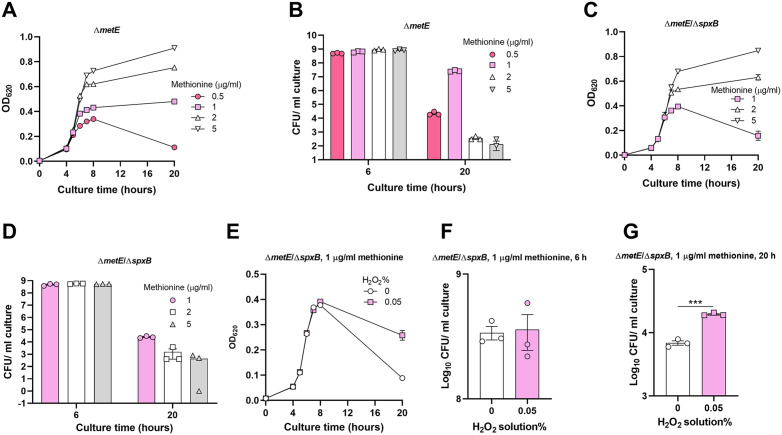
Inhibited autolysis by H_2_O_2_. **A,** Growth curves (OD_620_) and **B,** Survival (CFU) of Δ*metE* cultured in CDM with 0.5, 1, 2, or 5 μg/ml methionine. At 6 and 20 hr post inoculation, bacterial CFU was determined. **C,** Growth curves (OD_620_) and **D,** Survival (CFU) of Δ*metE*/Δ*spxB* cultured in CDM with 1, 2, or 5 μg/ml methionine. At 6 and 20 hr post inoculation, bacterial CFU was determined. **E,** Growth curves (OD_620_), **F,** Survival (CFU, 6 hr post inoculation) and **G,** Survival (CFU, 20 hr post inoculation) of Δ*metE*/Δ*spxB* cultured in CDM with 1 μg/ml methionine and 0 or 0.05% H_2_O_2_ solution. At 6 hr post inoculation, H_2_O_2_ solution was added. Each experiment was conducted in triplicate samples. P values < 0.001 (***).

How H_2_O_2_ contributed to bacterial survival under methionine starvation was still a mystery. **Fig 1D and 1E** showed that elevating intracellular pH by NH_3_.H_2_O attenuated bacterial survival. Bacteria underwent obvious autolysis in this condition. Fig 3E-3G show that reduced intracellular H_2_O_2_ level by *spxB* deletion attenuated bacterial survival. Bacteria also underwent obvious autolysis in this condition. These data indicate that increased H_2_O_2_ production by intracellular acidification inhibit autolysis to enhance bacterial survival under methionine starvation. To verify this, we firstly determined whether methionine starvation can induce autolysis. Δ*metE* was cultured in CDM with different concentration of methionine. Bacterial autolysis did not happen with 1 μg/ml methionine. A higher concentration (2 or 5 μg/ml) of methionine did not induce bacterial autolysis either. Interestingly, extremely low concentration (0.5 μg/ml) of methionine induced bacterial autolysis. The OD_620_ value of bacteria dropped from 0.34 to 0.11 (Fig 5A). At 6 hr post inoculation, the CFUs of bacteria cultured with different concentrations of methionine were almost consistent. However, at 20 hr post inoculation, the CFU of bacteria cultured with 0.5 μg/ml methionine was only 0.085% of the culture with 1 μg/ml methionine (Fig 5B). Bacteria were not starved with more methionine supplement (2 or 5 μg/ml), but bacterial CFU decreased sharply (Fig 5B). To exhibit the role of *spxB* in bacteria autolysis, Δ*metE*/Δ*spxB* was cultured in CDM with 1, 2, or 5 μg/ml methionine. Similar to Δ*metE*, neither 2 nor 5 μg/ml methionine induced bacterial autolysis. However, Δ*metE*/Δ*spxB* underwent obvious autolysis when cultured with 1 μg/ml methionine (Fig 5C) and its CFU was only 0.098% of Δ*metE* (Fig 5D).

The data above show that methionine starvation induces pneumococcal autolysis. The presence of *spxB* could not stop bacterial autolysis in extreme starvation of methionine (Δ*metE* cultured with 0.5 μg/ml methionine). However, in a moderate starvation (Δ*metE* cultured with 1 μg/ml methionine), the presence of *spxB* inhibited bacterial autolysis. To determine whether H_2_O_2_ produced by SpxB inhibits autolysis to enhance bacterial survival, Δ*metE*/Δ*spxB* was cultured in CDM with 1 μg/ml methionine and no or 0.05% H_2_O_2_ solution. Supply of 0.05% H_2_O_2_ solution at 6 hr post inoculation reduced bacterial autolysis (**Fig 5E**). Without H_2_O_2_ addition, the OD_620_ value dropped from 0.378 at 8 hr post inoculation to 0.088, dropping by 76.7%. However, with addition of H_2_O_2_ addition, the OD_620_ value dropped from 0.391 to 0.258, dropping by only 34% (**Fig 5E**). Before H_2_O_2_ addition, bacterial CFUs were consistent (**Fig 5F**). However, at 20 hr post inoculation (14 hr post H_2_O_2_ addition), the CFU of bacteria with H_2_O_2_ addition was 2.8-fold of the culture without H_2_O_2_ addition (**Fig 5G**). These data show that H_2_O_2_ could inhibit bacterial autolysis to enhance bacterial survival under methionine starvation.

### Inhibition of Intracellular acidification by sodium oxamate attenuates pneumococcal survival under methionine starvation

**Fig 6:**
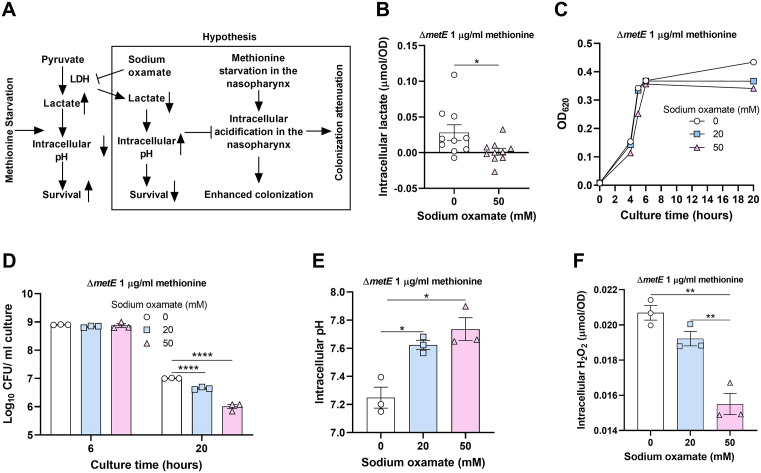
Inhibition of Intracellular acidification and survival attenuation by sodium oxamate. A, Hypothesis of the function of sodium oxamate. Under methionine starvation, supply of sodium oxamate inhibits lactate production by lactate dehydrogenase (LDH). Lactate production is then decreased, thus elevating intracellular pH. Therefore, bacterial survival is attenuated. Pneumococci undergo methionine starvation in the nasopharynx, which causes its intracellular acidification in the nasopharynx. This eventually enhances pneumococcal colonization. Sodium oxamate inhibits intracellular acidification of bacteria in the nasopharynx to attenuate pneumococcal colonization. **B,** Intracellular lactate (μmol/OD bacteria) of Δ*metE* cultured in CDM with 1 μg/ml methionine and supplied with 0- or 50 mM sodium oxamate. Each column has 10 samples. **C,** Growth curves (OD_620_), **D** Survival (CFU) and **E,** Intracellular pH of Δ*metE* cultured in CDM with 1 μg/ml methionine and supplied with 0-, 20-, or 50-mM sodium oxamate. At 6 and 20 hr post inoculation, bacterial CFU was determined. **F,** Intracellular H_2_O_2_ (μmol/OD bacteria) of Δ*metE* cultured in CDM with 1 μg/ml methionine and 0-, 20-, or 50 mM sodium oxamate. Each experiment was conducted in triplicate samples unless otherwise specified. P values < 0.05 (*), < 0.01 (**), and < 0.0001 (****).

Methionine is extremely shorted in the nasopharynx [11]. Therefore, we hypothesized that pneumococci undergo methionine starvation in the nasopharynx and this leads to its intracellular acidification, which eventually enhances pneumococcal colonization (**Fig 6A**). Based on this analysis, intracellular acidification could be a potential target to eliminate pneumococcal colonization. Lactate is produced from pyruvate by lactate dehydrogenase (LDH) in *S. pneumoniae* [26]. Sodium oxamate is an analog of pyruvate that inhibits the production of lactate [27]. We hypothesized that inhibited lactate production by sodium oxamate elevates intracellular pH and this attenuates bacterial survival (**Fig 6A**). Supply of sodium oxamate (50 mM) under methionine starvation decreased intracellular lactate level significantly, from 0.028 μmol/OD without sodium oxamate supply to 0.001 μmol/OD with 50 mM sodium oxamate supply (**Fig 6B**). Different from the increased OD_620_ value of the culture without sodium oxamate supply (from 0.368 at 6 hr to 0.434 at 20 hr), bacterial culture with 50 mM oxamate showed a decreased OD_620_ value (from 0.356 at 6 hr to 0.341 at 20 hr) (**Fig 6C**). Sodium oxamate supply caused bacterial autolysis. Bacterial survival was impaired significantly by sodium oxamate addition. At 6 hr post inoculation, bacterial CFU was almost consistent. However, at 20 hr post inoculation, the CFU of bacteria cultured with 20- or 50 mM sodium oxamate was 44.3% and 10.1% of the culture without sodium oxamate supply respectively (**Fig 6D**). Intracellular pH increased from 7.25 (no sodium oxamate) to 7.62 (20 mM sodium oxamate) and 7.73 (50 mM sodium oxamate) (**Fig 6E**). These data showed that sodium oxamate elevates intracellular pH by reducing lactate production to attenuate pneumococcal survival under methionine starvation. Addition of sodium oxamate successfully reduced intracellular H_2_O_2_ level under methionine starvation from 0.021 μmol/OD with no sodium oxamate to 0.019 μmol/OD with 20 mM sodium oxamate and 0.016 μmol/OD with 50 mM sodium oxamate (**Fig 6F**).

Again, to exclude the possible effect caused by *metE* deletion, wild-type strain (D39) was cultured in CDM with no methionine and 10 μg/ml cysteine. Sodium oxamate supply impaired bacterial growth (**S3A Fig**). At 20 hr post inoculation, bacterial CFU with 50 mM sodium oxamate supply reduced to 21.6% of the culture without sodium oxamate (**S3B Fig**). D39 showed cytoplasmic acidification (pH 7.20) at 9 hr post inoculation (**S3C Fig**). Supply of 20 mM or 50 mM sodium oxamate increased intracellular pH to 7.43 and 7.48 respectively at 9 hr post inoculation (**S3C Fig**). These data again confirmed the function of sodium oxamate for increasing bacterial intracellular pH to attenuate bacterial survival under methionine starvation.

### Intracellular acidification inhibitor (sodium oxamate) attenuates pneumococcal colonization

**Fig 7:**
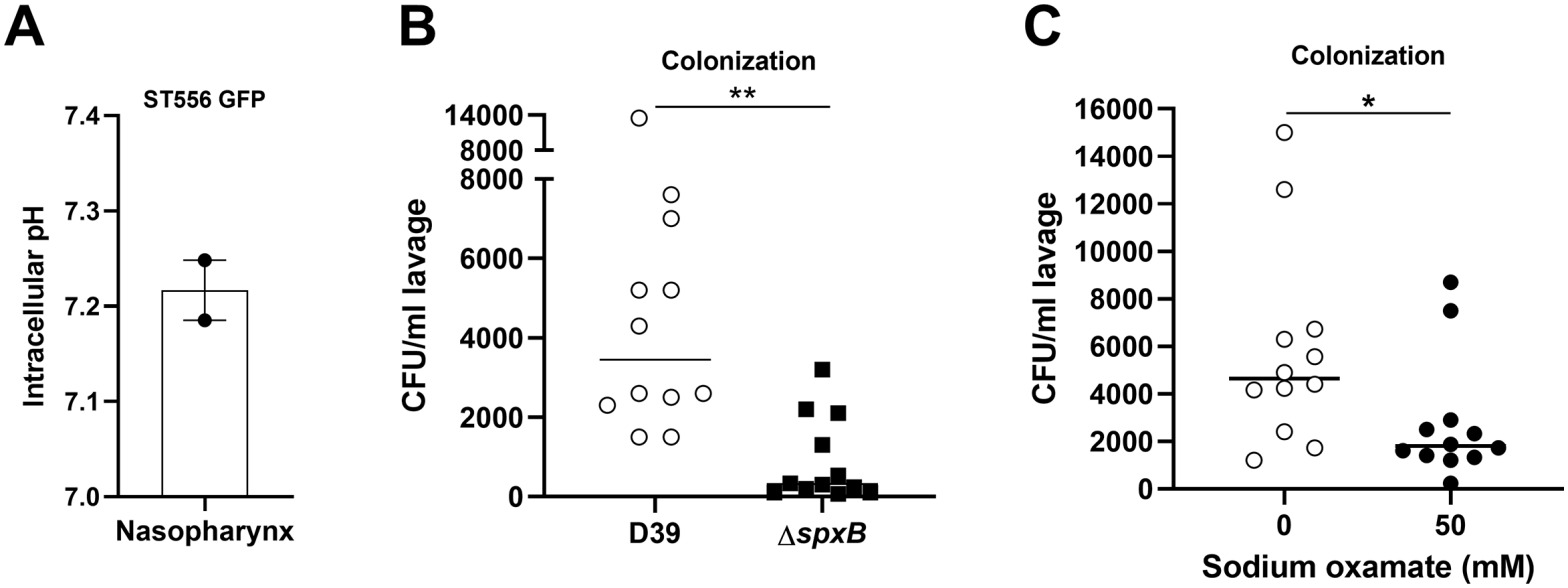
Colonization attenuation by intracellular acidification inhibitor (sodium oxamate). A, Intracellular pH of ST556 GFP. 12 hours after inoculation, bacteria were washed out from the nasopharynx. There are two samples. Each sample consisted of bacteria from the nasopharynx of 6 mice. **B,** Single colonization of WT (D39) and Δ*spxB*. Groups of CD1 mice (6-8 weeks old, n = 5-6) were intranasally inoculated with D39 (1 × 10^5^ CFU) or Δ*spxB* (1 × 10^5^ CFU). Two days after inoculation, bacteria were washed out from the nasopharynx to determine CFU. Each experiment was repeated at two different times. Horizontal lines show median values. **C,** Impact of sodium oxamate on D39 colonization. Groups of CD1 mice (6-8 weeks old, n = 5-6) were intranasally inoculated with D39 (1 × 10^5^ CFU). Two hours after inoculation, bacteria in the nasopharynx were supplied with 0- or 50-mM sodium oxamate to determine the impact of sodium oxamate on pneumococcal colonization. Each experiment was repeated at two different times. Horizontal lines show median values. P values < 0.05 (*) and < 0.01 (**).

We now confirmed that intracellular acidification could be targeted by sodium oxamate to attenuate pneumococcal survival under methionine starvation. To determine whether intracellular acidification could be a target for eliminating pneumococcal colonization, we firstly determined whether pneumococci undergo intracellular acidification during its colonization. We transformed the pIB166 plasmid [28] containing a GFP gene, the product of which is sensitive to pH, into bacteria to determine intracellular pH. For this method, to produce enough fluorescence signal, the number of viable bacteria cannot be too low. Due to the relatively low bacterial load in the nasopharynx for pneumococci D39 strain, we chose ST556 strain that has a higher bacterial load to replace D39 strain for determining the intracellular pH of *S. pneumoniae* during colonization. 5 μl bacterial liquid containing 5 × 10^7^ CFU ST556 with pH-sensitive GFP was dripped into each nasal cavity of mice. At 12 hr post inoculation, bacteria were collected from nasopharynx for determining intracellular pH. For each sample, the bacteria were collected from 6 mice. Consist with our hypothesis, the cytoplasm of ST556 was acidified to pH ~ 7.2 (**Fig 7A**).

In the colonization of a single strain, deletion of *spxB* almost eliminated pneumococcal colonization (**Fig 7B**), which emphasize the importance of H_2_O_2_ for pneumococcal survival in the nasopharynx. These data indicated that sodium oxamate attenuated pneumococcal colonization by reducing intracellular H_2_O_2_ level through inhibiting intracellular acidification. To determine whether sodium oxamate is able to eliminate colonization, 10 μl bacterial liquid containing 1 × 10^5^ CFU D39 was dripped into one nasal cavity of mice. After two hours’ colonization, 10 μl sodium oxamate (50 mM) was dripped into the same nasal cavity. This supply of sodium oxamate was done every one hour and lasted for 8 times. One hour after the last supply of sodium oxamate, bacteria were washed out from the nasopharynx for determining CFU. Addition of sodium oxamate into the nasopharynx did attenuate pneumococcal colonization. Bacterial colonization dropped to 38.7% of the control group (supply of Ringer’s solution) by this supply (**Fig 7C**). These data confirmed the function of sodium oxamate for impairing pneumococcal colonization.

### Intracellular acidification inhibitor (sodium oxamate) enhances the bactericidal effect of penicillin to pneumococci under methionine starvation

**Fig 8:**
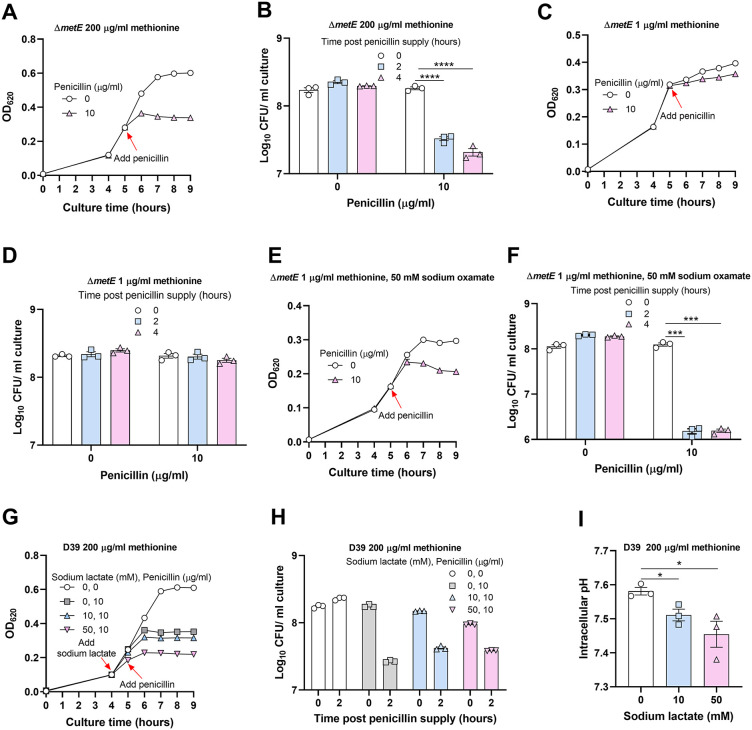
Enhanced penicillin killing of pneumococci under methionine starvation by sodium oxamate supply. **A,** Growth curves (OD_620_) and **B,** Survival (CFU) of Δ*metE* cultured in CDM with 200 μg/ml methionine and supplied with 0 or 10 μg/ml penicillin. Penicillin was added at 5 hr post inoculation. At 0, 2, and 4 hr post inoculation, bacterial CFU was determined. **C,** Growth curves (OD_620_) and **D,** Survival (CFU) of Δ*metE* cultured in CDM with 1 μg/ml methionine and supplied with 0 or 10 μg/ml penicillin. Penicillin was added at 5 hr post inoculation. At 0, 2, and 4 hr post inoculation, bacterial CFU was determined. **E,** Growth curves (OD_620_) and **F,** Survival (CFU) of Δ*metE* cultured in CDM with 1 μg/ml methionine and 50 mM sodium oxamate and supplied with 0 or 10 μg/ml penicillin. Penicillin was added at 5 hr post inoculation. At 0, 2, and 4 hr post inoculation, bacterial CFU was determined. **G,** Growth curves (OD_620_) and **H,** Survival (CFU) of D39 cultured in CDM with 200 μg/ml methionine and various concentrations of sodium lactate and penicillin. Sodium lactate was supplied at 4 hr post inoculation. Penicillin was added at 5 hr post inoculation. At 0 and 2 hr post penicillin supply, bacterial CFU was determined. **I,** Intracellular pH of D39 cultured in CDM with 200 μg/ml methionine and 0-, 10-, or 50-mM sodium lactate. Sodium lactate was supplied at 4 hr post inoculation. One hour later, bacterial intracellular pH was determined. Each experiment was conducted in triplicate samples. P values < 0.05 (*), < 0.001 (***), and < 0.0001 (****).

Antibiotics are effective substances for eliminating bacterial infection. For many bactericidal antibiotics, for example, the penicillin, it functions only when the bacteria are in an active state. Methionine starvation may down-regulate bacterial metabolic activity. Therefore, we hypothesized that methionine starvation inactivates the bactericidal function of penicillin. To test this hypothesis, we firstly confirmed the bactericidal function of penicillin by culturing Δ*metE* in a standard CDM (containing 200 μg/ml methionine) with or without penicillin supply. 10 μg/ml penicillin was added at 5 hr post inoculation. One hour after penicillin addition, bacterial growth was ceased (Fig 8A). Without penicillin supply, bacterial CFU was almost not changed from 5 hr to 9 hr post inoculation (Fig 8B). Conversely, at 2 hr and 4 hr post penicillin supply, bacterial CFU dropped to 18% and 11% of bacterial CFU before penicillin supply (0 hr) respectively (Fig 8B).

Interestingly, addition of penicillin under methionine starvation (Δ*metE*, 1 μg/ml methionine) did not cease bacterial growth (Fig 8C). Bacteria just grew more slowly. Without penicillin supply, bacterial CFU was almost not changed from 5 hr to 9 hr post inoculation (Fig 8D). Interestingly, penicillin supply did not kill bacteria significantly under methionine starvation (Fig 8D). Before penicillin supply, bacterial CFU was 2.1 × 10^8^. At 2 hr and 4 hr post penicillin supply, bacterial CFU was 2.0 × 10^8^ and 1.8 × 10^8^ respectively, which showed a tiny drop of CFU (Fig 8D). These data verified our hypothesis that penicillin does not kill ∆*metE* effectively under methionine starvation.

Methionine starvation induces intracellular acidification. Therefore, we hypothesized that intracellular acidification contributes to the inactivation of penicillin under methionine starvation and sodium oxamate addition recovers the function of penicillin by elevating intracellular pH. We firstly determined the impact of sodium oxamate addition on bacterial survival with penicillin treatment under methionine starvation. Δ*metE* was cultured in CDM with 1 μg/ml methionine and 50 mM sodium oxamate. At 5 hr post inoculation, 10 μg/ml penicillin was added. With penicillin supply, the OD_620_ value of bacterial culture had a slight drop (**Fig 8E**). Without penicillin supply (only supplied with sodium oxamate), bacterial CFU was almost not changed largely from 5 hr to 9 hr post inoculation (Fig 8F). Interestingly, in the culture with sodium oxamate and penicillin, bacterial CFU decreased sharply (Fig 8F). At 2 hr and 4 hr post penicillin supply, bacterial CFU dropped to 1.2% and 1.2% of the CFU of bacteria before penicillin supply (0 hr) respectively (Fig 8F). These data showed the enhanced bacteria-killing function of penicillin under methionine starvation by sodium oxamate addition.

We next verified the reduced bactericidal function of penicillin by intracellular acidification. D39 was cultured in CDM with sufficient methionine (200 μg/ml methionine). Addition of penicillin (10 μg/ml) at 5 hr post inoculation still ceased bacterial growth and killed bacteria effectively (Fig 8G and 8H). According to our previous work [17], sodium lactate is able to decrease intracellular pH. Addition of sodium lactate at 4 hr post inoculation further attenuated bacterial growth (Fig 8G). Interestingly, similar to methionine starvation, the supply of sodium lactate also reduced the bactericidal effect of penicillin. At 2 hr post penicillin supply, without sodium lactate supply, bacterial CFU dropped to 15.1% of bacterial CFU before penicillin supply (Fig 8H). However, with addition of 10- or 50-mM sodium lactate, this percentage increased to 28.2% and 40.6% respectively (Fig 8H), which shows the enhanced bacterial survival under penicillin treatment by sodium lactate supply. At one hour post sodium lactate addition (before penicillin supply), bacterial intracellular pH dropped from 7.58 (no sodium lactate supply) to 7.51 (10 mM sodium lactate supply) and 7.45 (50 mM sodium lactate supply) (Fig 8I). These data showed that intracellular acidification impairs the bactericidal function of penicillin.

In summary, we have the conclusion that methionine starvation inactivates the killing function of penicillin by intracellular acidification and sodium oxamate elevates intracellular pH under methionine starvation to enhance the bactericidal function of penicillin. Pneumococcal colonization is the first step of pneumococcal disease. In this work, bacteria collected from nasopharynx showed intracellular acidification. Therefore, we hypothesized that the bacteria-killing function of penicillin is restricted when it is used to eliminate pneumococcal colonization and sodium oxamate can recover the function of penicillin in this condition.

To improve the clinical/translational relevance of the findings, the enhanced killing of bacteria by the combination of penicillin and sodium oxamate was tested on ST556 strain that was isolated from an otitis media patient [29]. Culture of no methionine and 100 μg/ml cysteine created a methionine starvation condition (**S4A Fig**). At 6 hr post inoculation, there was no obvious difference between their CFUs (**S4B Fig**). However, at 20 hr post inoculation, the CFU of ST556 cultured with no methionine and 100 μg/ml cysteine was only 52.5% of the culture in standard CDM (S4B Fig). At 2 hr post penicillin addition (10 μg/ml, supplied at 6 hr post inoculation), the culture of ST556 in standard CDM experienced autolysis, while autolysis did not happen in the culture with no methionine and 100 μg/ml cysteine and its addition of 50 mM sodium oxamate (S4C Fig). At 2 hr post penicillin addition, bacterial CFU decreased to 45.5% of bacterial CFU before penicillin addition in the culture of no methionine and 100 μg/ml cysteine, while this ratio was 14.2% in the culture of standard CDM. This shows the impaired killing of penicillin under methionine starvation. However, the ratio of 45.5% decreased to 28.4% in the culture of no methionine, 100 μg/ml cysteine and 50 mM sodium oxamate (S4D Fig). This shows that supply of sodium oxamate enhanced killing by penicillin. Take together, the combination use of sodium oxamate and penicillin was also effective on the clinical strain ST556.

### Intracellular acidification inhibitor (sodium oxamate) plus penicillin strongly kills pneumococci in the nasopharynx

**Fig 9:**
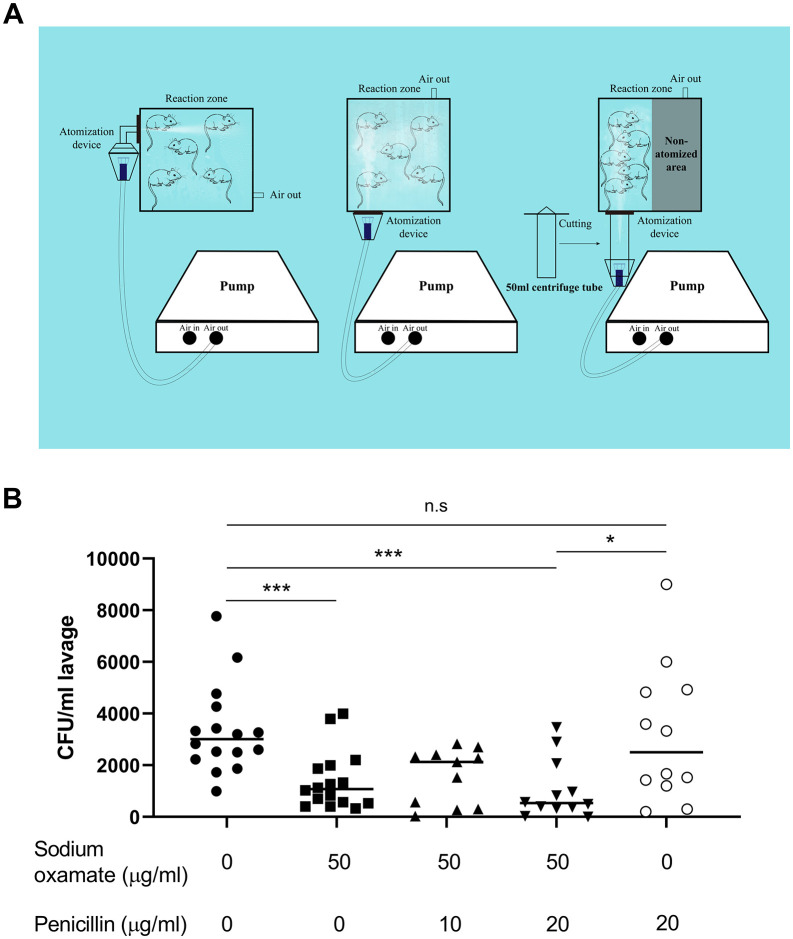
Strong elimination of pneumococcal colonization by combination of sodium oxamate and penicillin. **A,** Modified equipment for delivering drugs into the nasopharynx of mice. This equipment roughly consists of a pump, an atomization device and a container for accommodating mice. Originally, the atomization device consists of a spray nozzle, a lid, and a right-angle tube (left panel). In the first-time modification, the lid and the right-angle tube were removed and the container was rotated 90 degrees counterclockwise (middle panel). In the second-time modification, a bottom-cut 50 ml centrifuge tube was added between the container and the spray nozzle (right panel). Fig 9A was drawn by Adobe Illustrator 2020 by hand. **B,** Pneumococcal colonization with the supply of sodium oxamate and/or penicillin. Two hours after intranasal inoculation of D39 (1 × 10^5^ CFU), various concentrations of sodium oxamate and penicillin was delivered into the nasopharynx of mice by the modified equipment. After colonization, bacteria were washed out from the nasopharynx to determine CFU. Each experiment was repeated at two different times. Horizontal lines show median values. P values < 0.05 (*) and < 0.001 (***).

To determine the impact of penicillin or/and sodium oxamate addition on pneumococcal colonization in the nasopharynx, we modified an equipment to meet the demand of delivering drugs into the nasopharynx of mice efficiently (Fig 9A). The original nebulizer was brought from the company ZHONGSHI SCIENCE & TECHNOLOGY (Beijing, China), model number: ZS-DM-YWH. This equipment can roughly be divided into three parts, a pump providing air for atomizing, an atomization device to atomize the drugs, and a container for accommodating the mice and receiving the drugs (Fig 9A, left panel). The original atomization device consists of a spray nozzle, a lid, and a right-angle tube. Due to the lid and the right-angle tube, the produced fog was too thin to be delivered into the nasopharynx of mice. Most of the fog might be delivered into the lung of mice. Therefore, we removed the lid and the right-angle tube and rotated the container 90 degrees counterclockwise. Then we got the first-time modified equipment, in which the container gets the fog from bottom (Fig 9A, middle panel). Although the fog became obviously thicker, the time of consuming 45 ml liquid became obviously shorter (only several minutes). This could not meet the demand of making the drug delivered into the nasopharynx for a relatively long time. Therefore, we further modified this equipment. We cut the bottom of a 50 ml centrifuge tube and upended this cut tube in the atomization cup. The cut side was connected to the container. Besides, the volume of accommodating mice was narrowed to make the mice better get the fog. Then, we got the final version (Fig 9A, right panel). By this finally modified equipment, the spraying of consuming 45 ml liquid can last 20 min every time.

10 μl bacterial liquid containing 1 × 10^5^ CFU D39 was dripped into one nasal cavity of mice. After two hours’ colonization, 45 ml Ringer’s solution, sodium oxamate (dissolved in Ringer’s solution), penicillin (dissolved in Ringer’s solution), or sodium oxamate plus penicillin (dissolved in Ringer’s solution) was sprayed by this modified nebulizer equipment. Spraying was performed once in every hour and lasted for 4 times. At 6 hr post inoculation, bacteria were washed out from nasopharynx for determining CFU.

Fig 9B shows that the median value of bacterial CFU with Ringer’s solution supply was 3016/ml (control group). Spraying 50 mM sodium oxamate decreased bacterial CFU to 1083/ml (35.9% of the control group), which has a similar effect to the dripping of sodium oxamate into the nasopharynx of mice (38.7%). This result not only again verified the function of sodium oxamate for eliminating pneumococcal colonization, but also showed the high efficiency of delivering drugs into the nasopharynx of mice by this modified equipment. Surprisingly, bacterial CFU from nasopharynx sprayed with 10 μg/ml penicillin plus 50 mM sodium oxamate was even higher than sprayed with 50 mM sodium oxamate singly. We speculated that although 10 μg/ml penicillin is enough to kill bacteria in the *in vitro* culture, the nasopharynx cannot get the same amount of penicillin, therefore bacteria cannot be killed effectively. The concentration of penicillin was doubled (20 μg/ml) to determine this possibility. Excitingly, 20 μg/ml penicillin plus 50 mM sodium oxamate decreased bacterial colonization to only 17.7% of the control group (533 CFU/ml). Particularly, spraying 20 μg/ml penicillin singly did not attenuate pneumococcal colonization significantly. These data showed that penicillin alone is hard to eliminate pneumococcal colonization and elimination of colonization by sodium oxamate alone is limited. However, the combination of penicillin and sodium oxamate almost eliminated pneumococcal colonization.

## Discussion

Successful colonization in the nasopharynx is the first step for *S. pneumoniae* to elicit invasive disease. To impair pneumococcal colonization is theoretically a perfect way to prevent pneumococcal disease. Unfortunately, except for the vaccines, few efforts have been made to realize this aim. The long-term colonization of *S. pneumoniae* in the nasopharynx [20] is a fascinating phenomenon, which means *S. pneumoniae* has a strong surviving ability in the nasopharynx. Pneumococci need to attach to the epithelial cells [30], evade host immunity [31], particularly grow or survive on the mucosal surface where the nutrition is limited [32]. Knowing how *S. pneumoniae* survives in this nutrition-limited environment helps us find the targets for eliminating pneumococcal colonization.

In the work by Philips et al., glucose was even not detected in the nasal secretions of healthy volunteers (age ~ 23) [33]. However, pneumococci have a strong ability to utilize other carbohydrates in the nasopharynx. A total of 21 phosphotransferase systems, 7 ATP binding cassettes (ABC) and a single ATP binding cassette protein (ATPase) had been identified in pneumococci genome [34], accounting for over 30% of all transporters in pneumococci [35]. This enables *S. pneumoniae* to utilize at least 32 carbohydrates [36]. Besides, neuraminidase A of pneumococci cuts sialic acid from the surface glycoconjugates of epithelial cells [37] and the cut sialic acid can be imported by the corresponded transporter, which enhances pneumococcal colonization [38].

Amino acids are important nutrients, especially methionine. Our work shows that *S. pneumoniae* undergoes methionine starvation in the nasopharynx. During pneumococcal colonization, methionine starvation leads to intracellular acidification of *S. pneumoniae*, which enhances pneumococcal colonization. Intracellular acidification was verified to be a target for eliminating pneumococcal colonization in this work. However, how intracellular acidification enhances bacterial survival was necessarily to be explained. In this work, a surprising phenomenon was found that increased intracellular H_2_O_2_ level by intracellular acidification enhanced bacterial survival under methionine starvation.

*S. pneumoniae* is an aerotolerant anaerobic bacterium. It is exposed to different concentrations of oxygen in different host niches, from extremely low concentration in the blood, to around 5% in the lower respiratory tract, to near 20% on top of the nasopharynx [21]. This makes *S. pneumoniae* be able to utilize the oxygen to produce large amount of H_2_O_2_ by its pyruvate oxidase SpxB^21^. The H_2_O_2_ produced by *S. pneumoniae* causes damage to other bacteria in the upper respiratory tract, such as *Haemophilus influenzae* and *Neisseria meningitidis* [39], which may provide a competitive advantage for pneumococcal colonization. Despite this benefit, H_2_O_2_ causes threat to the membrane homeostasis of pneumococci by affecting fatty acid unsaturation [40]. However, *S. pneumoniae* does not have catalase to decompose H_2_O_2_. It has been revealed that *S. pneumoniae* defends against reactive oxygen species (ROS) by at least three strategies. The enzyme detoxification mechanisms remove ROS by some enzymes, such as NADH oxidase that converts O_2_ to H_2_O [41], and a putative alkyl hydroperoxidase that converts peroxide compounds to water and alcohol [42]. The enzymatic repair mechanisms protect pneumococci against ROS by some enzymes, for example, the HtrA that reduces the sensitivity of pneumococci to H_2_O_2_ [43]. The cations can detoxify ROS and act as a signal for gene regulation to defend against ROS, for example, the Mn^2+^ in *S. pneumoniae* [44].

**Fig 10:**
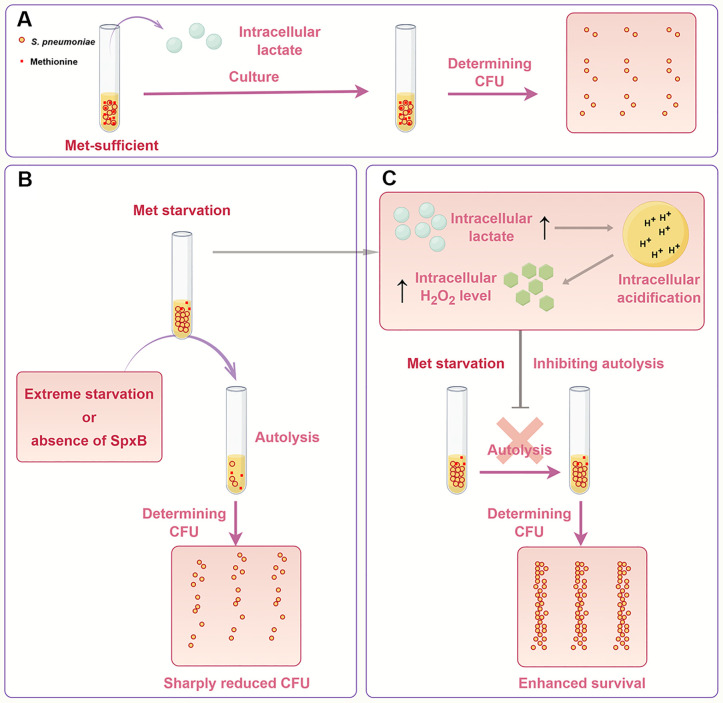
Diagram of the enhanced survival under methionine starvation by elevated intracellular H_2_O_2_ level. **(By Figdraw.)**. **A,** In the culture with sufficient methionine (Met), bacterial cytoplasm is not acidified due to low intracellular lactate level. The CFU is low in this culture. **B,** Under methionine starvation, bacterial autolysis happens in the absence of *spxB* or extreme shortage of methionine. The CFU is low in this culture. **C,** Under methionine starvation, increased intracellular lactate level leads to intracellular acidification, which promotes H_2_O_2_ production by SpxB. A higher H_2_O_2_ level inhibits bacterial autolysis to enhance bacterial CFU (a high CFU). The website of Figdraw (an online platform) is https://www.figdraw.com/static/index.html#/.

H_2_O_2_ must play extremely important role for pneumococcal survival in the host niches, otherwise *S. pneumoniae* won’t produce large amount of H_2_O_2_ and have so many genes to defend against H_2_O_2_. The benefits would not be restricted to competitive advantage in the upper respiratory tract. In this work, H_2_O_2_ was found to enhance pneumococcal survival under methionine starvation. Interestingly, intracellular acidification increased the level of intracellular H_2_O_2_. To our best knowledge, it is the first time that H_2_O_2_ was reported to benefit bacterial survival in the *in vitro* single culture. How it works was illustrated in **Fig 10**. With sufficient methionine, lactate production is limited and bacterial cytoplasm is not acidified. Bacterial survival is therefore poor (low number of CFU) (Fig 10A). In the absence of SpxB (low intracellular H_2_O_2_ level) or extreme shortage of methionine, methionine starvation induces bacterial autolysis. Once the autolysis happens, bacterial CFU would sharply be reduced (Fig 10B). However, *S. pneumoniae* has the mechanism to defend against autolysis. Methionine starvation increased intracellular lactate level, which acidified bacterial cytoplasm. This intracellular acidification promotes H_2_O_2_ production by SpxB. The increased intracellular H_2_O_2_ level inhibits bacterial autolysis (Fig 10C). Therefore, a relatively high level of CFU was maintained under methionine starvation. Bacterial survival is enhanced by this way.

**Fig 11:**
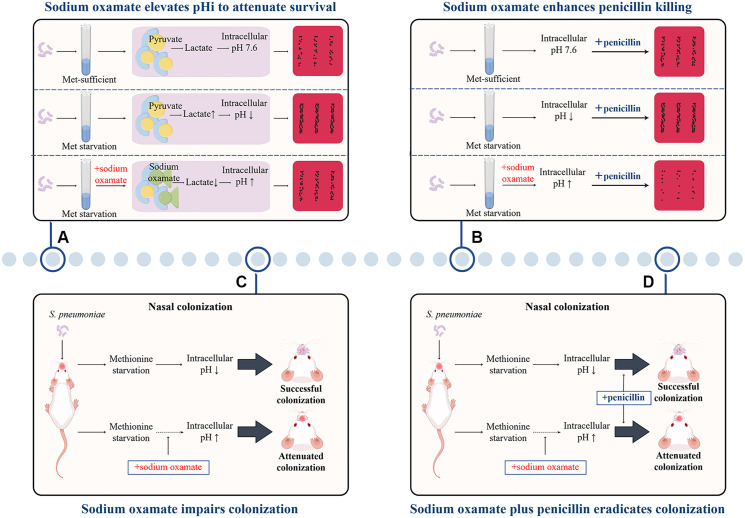
Diagram of the function of sodium oxamate for attenuating pneumococcal survival and eliminating pneumococcal colonization. **(By Figdraw.). A,** Sodium oxamate elevates intracellular pH (pHi) to attenuate survival. Lactate production from pyruvate is enhanced and bacterial intracellular pH decreased due to methionine starvation, which leads to enhanced bacterial survival. Sodium oxamate supply inhibits lactate production and elevates bacterial intracellular pH under methionine starvation, leading to attenuated bacterial survival. **B,** Sodium oxamate enhances penicillin killing. Intracellular acidification under methionine starvation inhibits the killing function of penicillin. Sodium oxamate supply elevates intracellular pH under methionine starvation, leading to enhanced killing of bacteria by penicillin. **C,** Sodium oxamate enhances penicillin killing. Intracellular acidification enhances pneumococcal colonization. Sodium oxamate supply elevates pneumococcal intracellular pH during colonization, which attenuates pneumococcal colonization. **D,** Sodium oxamate plus penicillin eradicates colonization. Penicillin alone cannot eliminate pneumococcal colonization. Sodium oxamate supply elevates bacterial intracellular pH during colonization and bacteria become sensitive to penicillin treatment in the nasopharynx. Simultaneous use of sodium oxamate and penicillin eliminates pneumococcal colonization more effectively than single use of sodium oxamate or penicillin. The website of Figdraw is https://www.figdraw.com/static/index.html#/.

Intracellular acidification plays a central role in this survival enhancement under methionine starvation, which makes it a potential drug target. In this work, sodium oxamate was used to target intracellular acidification to attenuate pneumococcal survival and eliminate pneumococcal colonization (summarized in Fig 11). Fig 11A: When bacteria are not under methionine starvation, the production of lactate from pyruvate by LDH is limited. Therefore, bacterial intracellular pH is ~ 7.6. Bacteria survival ability is not strong in this condition (the CFU is not high). When bacteria are under methionine starvation, lactate production was up-regulated. Therefore, bacterial intracellular pH decreased. This intracellular acidification enhances bacterial survival (the CUF is high). Sodium oxamate supplied under methionine starvation competes with pyruvate to reduce lactate production. This elevates intracellular pH. Bacterial survival was significantly attenuated in this condition (reduced CFU). Fig 11B: When bacteria are not under methionine starvation, penicillin treatment kills bacteria significantly due to the normal intracellular pH (~ 7.6). However, under methionine starvation, treatment with penicillin does not kill bacterial effectively due to intracellular acidification. Interestingly, under methionine starvation, penicillin treatment kills bacteria effectively due to the elevated intracellular pH by sodium oxamate supply. Fig 11C: During pneumococcal colonization, methionine starvation leads to intracellular acidification of *S. pneumoniae*, which enhances pneumococcal colonization. Supply of sodium oxamate in the nasopharynx elevates pneumococcal intracellular pH. Therefore, pneumococcal colonization was attenuated. **Fig 11D**: During pneumococcal colonization, penicillin treatment does not eradicate pneumococcal colonization effectively due to the intracellular acidification under methionine starvation. Interestingly, penicillin treatment almost eradicates pneumococcal colonization due to the elevated intracellular pH by sodium oxamate supply in the nasopharynx.

As an analog of pyruvate, sodium oxamate has been demonstrated to inhibit lactate dehydrogenase directly to reduce lactate production [27,45–47]. Some biological functions of sodium oxamate have been explored. Enhanced glycolysis, liposynthesis and lipolysis by sodium oxamate supply benefit the growth and glucose homeostasis of *Micropterus salmoides* fed with high-carbohydrate diets [27]. Elevated plasma lactate level was associated with type 2 diabetes and markers of insulin resistance [48,49]. A higher expression level of LDH-A was observed in the islet cells of *db*/*db* mice^46^. Sodium oxamate treatment reduced the expression of LDH-A [46]. Intraperitoneal injection of oxamate decreases blood glucose level, increase insulin secretion and insulin sensitivity of *db*/*db* mice [46]. Therefore, oxamate is a potential drug for type 2 diabetes treatment. Taxol (paclitaxel) is a drug that treats human breast cancer by inducing apoptosis of breast cancer cells [50]. Taxol-resistant subclones of breast cancer cells exhibit an up-regulated expression and activity of LDH-A [47]. Inhibition of LDH-A by oxamate makes Taxol-resistant cells sensitive to Taxol again^47^. This combination of oxamate and Taxol is a potential therapy for treatment of breast cancer with Taxol-resistant cells [47].

The studies above show the function of oxamate for regulating LDH expression, lactate production, and glucose metabolism. Oxamate has been a potential drug for treatment of type 2 diabetes and breast cancer with Taxol-resistant cells. To our best knowing, it is the first time that oxamate was reported to increase bacterial intracellular pH under starvation. This provides a brand-new perspective for treating bacterial infection or eliminating bacterial colonization related to bacterial intracellular acidification. There are some other bacteria that colonize in the nasopharynx. Successful colonization is also essential for them to cause disease. *Staphylococcus aureus* isolated from the blood of patients with *S. aureus* bacteremia was identical to the *S. aureus* isolated from anterior nares, which shows the contribution of colonization to bacteremia caused by *S. aureus* [51]. In a neonatal colonization model, a single organism of *Haemophilus influenzae* type b caused bacteremia [52]. Intracellular acidification may also occur in these bacteria during their colonization in the nasopharynx.

In summary, there are two major findings in this work. Firstly, we illustrated how intracellular acidification was realized and how it enhances bacterial survival by promoting H_2_O_2_ production. Secondly, we successfully targeted intracellular acidification by sodium oxamate to impair pneumococcal survival and colonization. A novel therapy that combines sodium oxamate and penicillin to eliminate pneumococcal colonization was provided in this work. Our work provides a novel way of eradicating pneumococcal colonization by targeting intracellular acidification.

## Materials and methods

### Ethics statement

All animal infection procedures were carried out according to an animal protocol approved by the Institutional Animal Care and Use Committee of Lishui University. The approval number is 2025D053.

### Bacterial cultivation and reagents

In this work, *S. pneumoniae* serotype 2 (D39) [53] was used as the parental strain. Pneumococci were cultured in Todd-Hewitt broth with 0.5% yeast extract (THY), chemically defined medium (CDM) or tryptic soy agar (TSA) plates with 4% sheep blood at 37°C, 5% CO_2_ as previously described [54]. CDM was prepared based on previous study [55]. Antibiotics were supplemented to the media when required as described [54]. All chemicals and enzymes were brought from Sigma (Beijing, China) and New England BioLabs (Beijing, China), respectively. All strains used in this work are described in S1 Table.

### Mutant construction

Promoter replacement of *ldh* with the promoter of SPD0818 was operated in TH4306, a streptomycin-resistant derivative of strain D39 or its *metE* deletion strain (Δ*metE*) from previous work [15] by natural transformation using Janus cassette (JC)-based counter selection as previously described [56,57]. Briefly, the up- (amplified by Pr0029 and Pr0030) and down-stream (amplified by Pr0031 and Pr0032) sequences of the promoter of *ldh*, and JC were individually amplified. The amplicons were linked by enzymatic digestion and ligation. For natural transformation, the promoter of *ldh* was replaced by JC, which contains the kanamycin resistance gene *kan* for selection and *rpsL* for counter-selection. The transformants were selected by kanamycin resistance. For counter-selection, the upstream of *ldh* promoter was amplified by Pr0029 and Pr0037, the promoter of SPD0818 (250 bp upstream of the ATG codon of SPD0818) was amplified by Pr0038 and Pr0039, and the downstream of *ldh* promoter was amplified by Pr0040 and Pr0032. The amplicons were digested with BsaI and fused as described [56]. Deletion of *spxB* gene was operated in TH4306 (D39) or Δ*metE*. The up- (amplified by Pr14761 and Pr14762) and down-stream (amplified by Pr14763 and Pr14764) sequences of *spxB* gene and JC were individually amplified. The amplicons were linked by enzymatic digestion and ligation. For counter-selection, the upstream of *spxB* was amplified by Pr14761 and Pr14765, and the downstream of *spxB* was amplified by Pr14766 and Pr14764. The amplicons were digested with BsaI and fused. Deletion of *lctO* gene was operated in Δ*metE* or Δ*metE*/Δ*spxB*. The up- (amplified by Pr0053 and Pr0054) and down-stream (amplified by Pr0055 and Pr0056) sequences of *lctO* gene and JC were individually amplified. The amplicons were linked by enzymatic digestion and ligation. For counter-selection, the upstream of *lctO* was amplified by Pr0053 and Pr0057, and the downstream of *spxB* was amplified by Pr0058 and Pr0056. The amplicons were digested with BsaI and fused. The primers used are listed in S2 Table.

### Characterization of bacterial growth and survival

Growth of pneumococci was determined as previously described [58]. Briefly, bacteria were grown in THY to an optical density at 620 nm (OD_620_) of 0.5. Then bacteria were washed twice with Ringer’s solution by centrifugation and resuspension. Bacterial pellets were resuspended in Ringer’s solution to OD_620_ 0.5, and then diluted at a 1:100 ratio in CDM with complete contents or CDM with various modifications in amino acid content. Bacterial growth was measured by determining the value of OD_620_. Each point of growth curves represents the mean OD_620_ value of three replicates at each time point. Most error bars of the growth curves were too short to be exhibited (also for other growth data in this paper).

### Intracellular pH determination

Intracellular pH was determined using a pH-sensitive green fluorescent protein (pH-GFP) as previously described [16,28]. Briefly, plasmid pIB166 harboring the pH-GFP gene was transformed into pneumococci by natural transformation. Strains with pH-GFP plasmid were cultured in THY till mid-log phase, then washed and diluted into CDM as in bacterial growth determination. At the time for pH detection, 0.6 OD_620_ bacteria were collected and washed by colorless CDM prepared as previously described [59]. Bacterial pellets were resuspended in 1200 μl colorless CDM and dispensed into black 96-well plates (200 μl/well) (Corning Incorporated, USA). Fluorescence was determined at Reading1 (excitation 395 at nm, emission at 510 nm) and Reading2 (excitation at 475 nm, emission at 510 nm) by a microplate reader (BioTek Synergy H1, Agilent, USA). The ratio of “Reading1-Blank1” to “Reading2-Blank2 (X) was used to determining the value of intracellular pH (Y) with an equation: Y = 2.1868 × ln(X) + 7.0626.

### Intracellular lactate determination

Intracellular lactate level was determined by a lactic acid content assay kit (Solarbio, Beijing, China). Briefly, 0.5 OD bacteria cultured in CDM (1-1.5 ml) were collected and then washed twice with Ringer’s solution by centrifugation and resuspension. Bacterial pellets were resuspended in 50 μl Ringer’s solution with 0.2% sodium deoxycholate and incubated at 37°C for 8 min for lysis. 200 μl extracting solution 1 was then added, mixed and centrifuged to get the supernatant. 200 μl supernatant was collected and mixed with 30 μl extracting solution 1. The following steps are operated according to the giving protocol of this kit. The standard curve for determining the intracellular lactate content is Y = 0.924X + 0.0176. Y is the intracellular lactate content (μmol/OD). X is the OD_570_ value.

### qRT-PCR

Quantitative real-time reverse transcriptase PCR (qRT-PCR) was performed as described [60]. Briefly, Δ*metE* or Δ*metE* P*ldh*::P0818 was diluted in duplicate of 7 ml CDM supplemented with various concentrations of methionine and glutamine. At 6 hr post inoculation, bacteria were collected and processed for extraction of total RNA using RNAprep Kit (Tiangen, Beijing). Extracted RNA was used to construct cDNA pools with iScript cDNA Synthesis Kit (Bio-Rad, USA). Pr0041 and Pr0042 were used to amplify *ldh*. SPD0857 (*era*, amplified by Pr7932 and Pr7933) was used as a reference gene for normalization of gene expression. The primer sequences are listed in S2 Table. Each gene was tested using triplicate samples for the first time, and subsequently retested once.

### Intracellular H_2_O_2_ determination

Intracellular H_2_O_2_ level was determined by a H_2_O_2_ content assay kit (Beyotime Biotechnology, Shanghai, China). Briefly, 0.2 OD bacteria cultured in CDM were collected and then washed once with Ringer’s solution by centrifugation and resuspension. Bacteria pellets were resuspended in 50 μl Ringer’s solution with 0.2% sodium deoxycholate and incubated at room temperature for 15 min for lysis. 100 μl lysis buffer in the kit was then added, mixed and centrifuged to get the supernatant. 100 μl supernatant was collected for determination. The following steps are operated according to the giving protocol of this kit. The standard curve for determining the intracellular H_2_O_2_ content is Y = 0.0216X - 0.00672. Y is the H_2_O_2_ content (μmol/OD). X is the OD_560_ value.

### Animal experiments

All mouse experiments were carried out in female CD1 mice (6–8 weeks old, PIZHOU Oriental, Xuzhou, China). Pneumococcal carriage was assessed as previously described [15] with minor modification. Briefly, mice were infected by intranasal inoculation of D39 either by dripping or spraying. The residing bacteria in the nasopharynx were estimated by washing the nasal passage with 1 ml of Ringer’s solution and determining CFU in lavage samples by culturing bacteria on TSA blood plates. For each group, 5–6 mice were used. For measuring the intracellular pH of pneumococci colonized in the nasopharynx, 5 μl bacterial liquid containing 5 × 10^7^ CFU ST556 with pH-sensitive GFP was dripped into each nasal cavity of mice. At 12 hr post inoculation, bacteria were collected from the nasopharynx of mice for measuring intracellular pH. For each sample, the bacteria were collected from 6 mice. For determining the importance of *spxB* gene for colonization, single strain colonization of D39 or Δ*spxB* was operated. 10 μl bacterial liquid containing 1 × 10^5^ CFU was dripped into one nasal cavity of mice. For determining the effect of sodium oxamate for eliminating pneumococcal colonization, 10 μl bacterial liquid containing 1 × 10^5^ CFU D39 was dripped into one nasal cavity of mice. 2 hours after inoculation, 10 μl sodium oxamate (50 mM) was dripped into the nasal cavity. This supply of sodium oxamate was done every one hour and lasted for 8 times. One hour after the last supply of sodium oxamate, bacteria were collected for determining CFU. For determining the function of sodium oxamate or penicillin on eradicating colonization by the way of spraying, 10 μl bacterial liquid containing 1 × 10^5^ CFU D39 was dripped into one nasal cavity of mice. 2 hours after inoculation, 45 ml Ringer’s solution, sodium oxamate (dissolved in Ringer’s solution), penicillin (dissolved in Ringer’s solution), or sodium oxamate plus penicillin (dissolved in Ringer’s solution) was sprayed. Sparing was performed once every hour and lasted for 4 times. At 6 hr post inoculation, bacteria were collected from nasopharynx for determining CFU.

### Statistical analysis

All experiments reported in this work were conducted in triplicate samples and repeated at least once. The relevant data are presented as mean ± SEM (standard error of mean), and analyzed by two-tailed unpaired Student’s *t* test in Graphpad Prism 8. Significant differences are defined by *P* values of < 0.05 (*), < 0.01 (**), < 0.001 (***), and < 0.0001 (****).

## Supporting information

S1 Fig Survival (CFU, 6 hr post inoculation) of Δ*metE* cultured in CDM with 200 μg/ml methionine before H_2_O_2_ solution. At 6 hr post inoculation, H_2_O_2_ solution was added. Each experiment was conducted in triplicate samples.
(TIF)

S2 Fig Importance of *lctO* for pneumococcal survival under methionine starvation. A, Growth curves (OD_620_) and B, Survival (CFU) of Δ*metE*/Δ*spxB*, Δ*metE*/Δ*lctO* and Δ*metE*/Δ*spxB*/Δ*lctO* cultured in CDM with 1 μg/ml methionine. At 8 and 20 hr post inoculation, bacterial CFU was determined. Each experiment was conducted in triplicate samples. P values < 0.0001 (****).
(TIF)

S3 Fig Inhibition of intracellular acidification and survival attenuation by sodium oxamate in D39 strain. A, Growth curves (OD_620_), B, Survival (CFU, 20 hr post inoculation) and C, Intracellular pH (9 hr post inoculation) of D39 cultured in CDM with no methionine and 10 μg/ml cysteine and supplied with 0-, 20-, or 50-mM sodium oxamate. Each experiment was conducted in triplicate samples. P values < 0.05 (*) and < 0.01 (**).
(TIF)

S4 Fig Enhanced penicillin killing of ST556 strain of pneumococci under methionine starvation by sodium oxamate supply. A, Growth curves (OD_620_) and B, Survival (CFU) of ST556 cultured in CDM with no methionine and 100 μg/ml cysteine or standard CDM. C, Growth curves (OD_620_) and D, Survival (CFU) of ST556 cultured in CDM with no methionine and 100 μg/ml cysteine, no methionine, 100 μg/ml cysteine and 50 mM sodium oxamate, or standard CDM. Penicillin was added at 6 hr post inoculation. At 0 and 2 hr post inoculation, bacterial CFU was determined. Each experiment was conducted in triplicate samples. P values < 0.01 (**).
(TIF)

S1 Table Bacterial strains used in this study. (DOCX)

S2 Table Primers used in this study. (DOCX)

S1 Data Data. (XLSX)
