# Supplementary figures and images for: Eliminate pneumococcal colonization by targeting intracellular acidification that promotes H2O2 production to enhance bacterial survival
Source: PLoS Pathog. 2026 Jun 23;22(6):e1014381. doi: 10.1371/journal.ppat.1014381 (PMC13313337; doi:10.1371/journal.ppat.1014381)

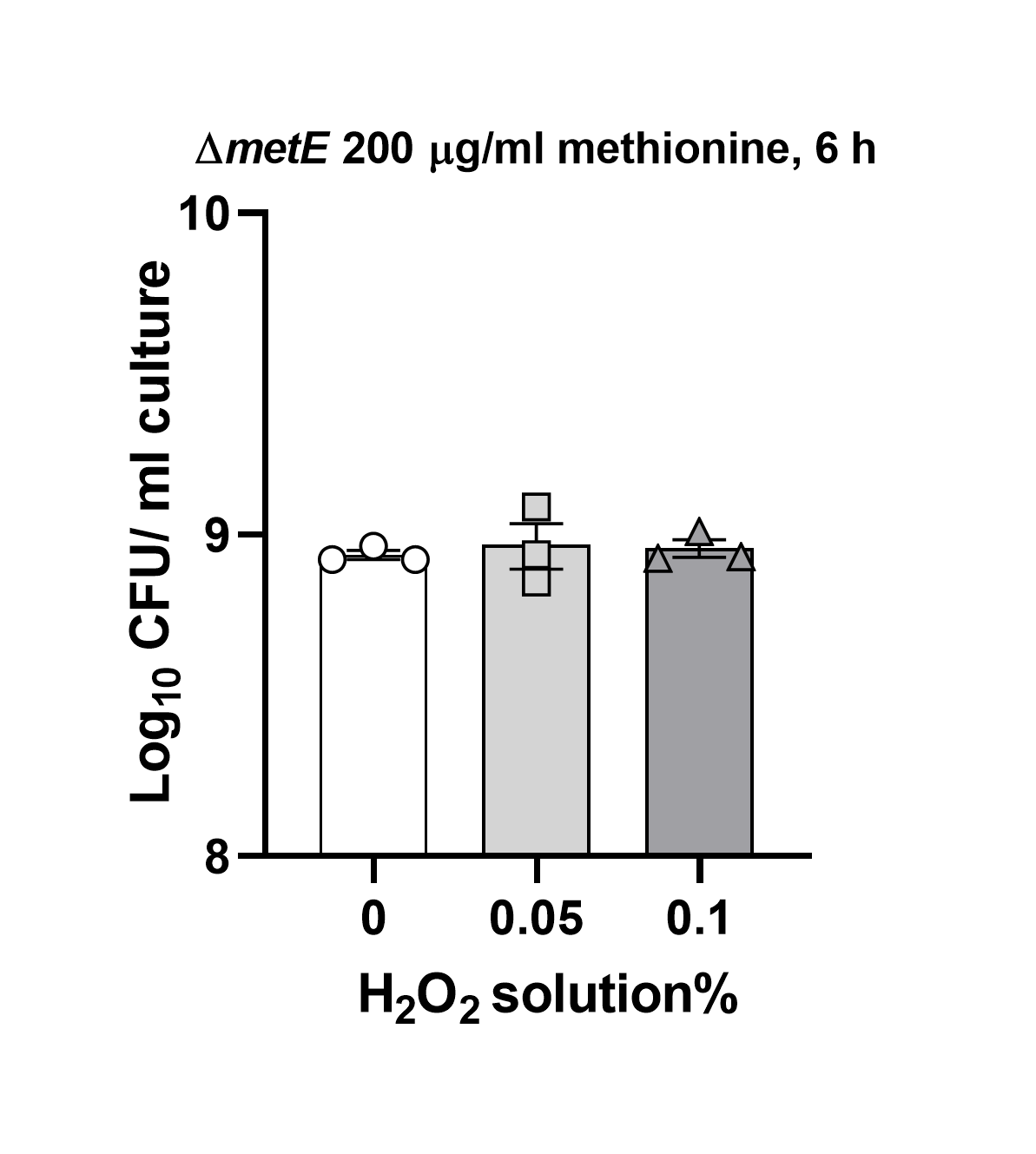

Supplement: S1 Fig — At 6 hr post inoculation, H2O2 solution was added. Each experiment was conducted in triplicate samples. (TIF) [file ppat.1014381.s001.tif]

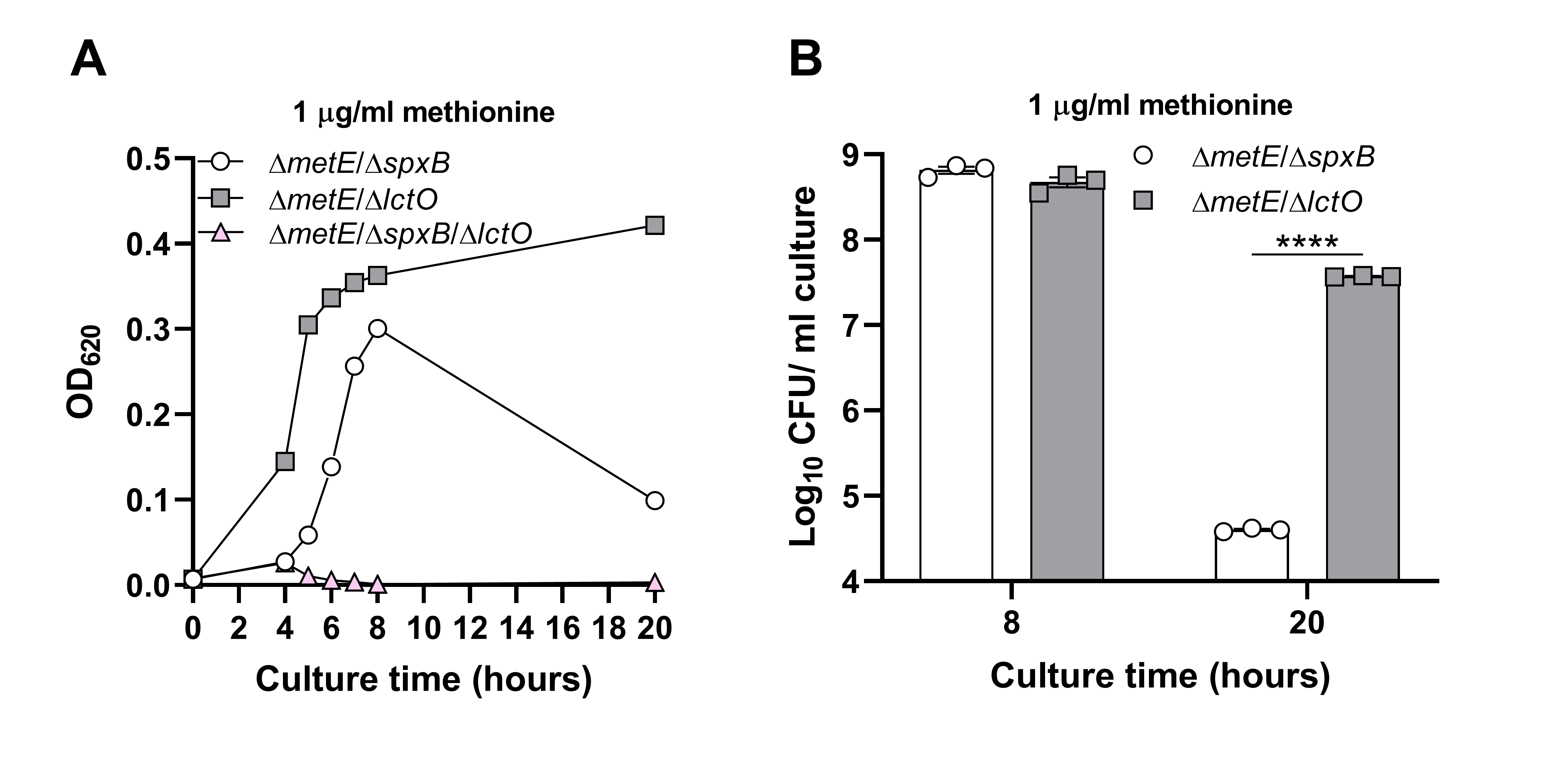

Supplement: S2 Fig — A, Growth curves (OD620) and B, Survival (CFU) of ΔmetE/ΔspxB, ΔmetE/ΔlctO and ΔmetE/ΔspxB/ΔlctO cultured in CDM with 1 μg/ml methionine. At 8 and 20 hr post inoculation, bacterial CFU was determined. Each experiment was conducted in triplicate samples. P values < 0.0001 (****). (TIF) [file ppat.1014381.s002.tif]

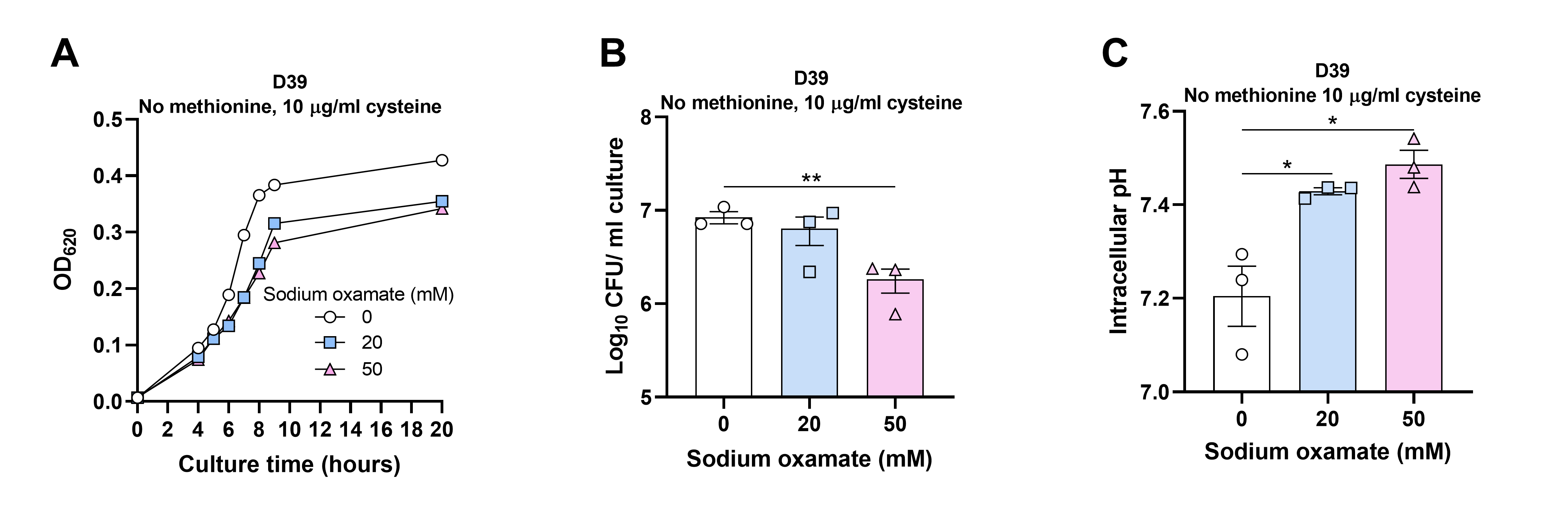

Supplement: S3 Fig — A, Growth curves (OD620), B, Survival (CFU, 20 hr post inoculation) and C, Intracellular pH (9 hr post inoculation) of D39 cultured in CDM with no methionine and 10 μg/ml cysteine and supplied with 0-, 20-, or 50-mM sodium oxamate. Each experiment was conducted in triplicate samples. P values < 0.05 (*) and < 0.01 (**). (TIF) [file ppat.1014381.s003.tif]

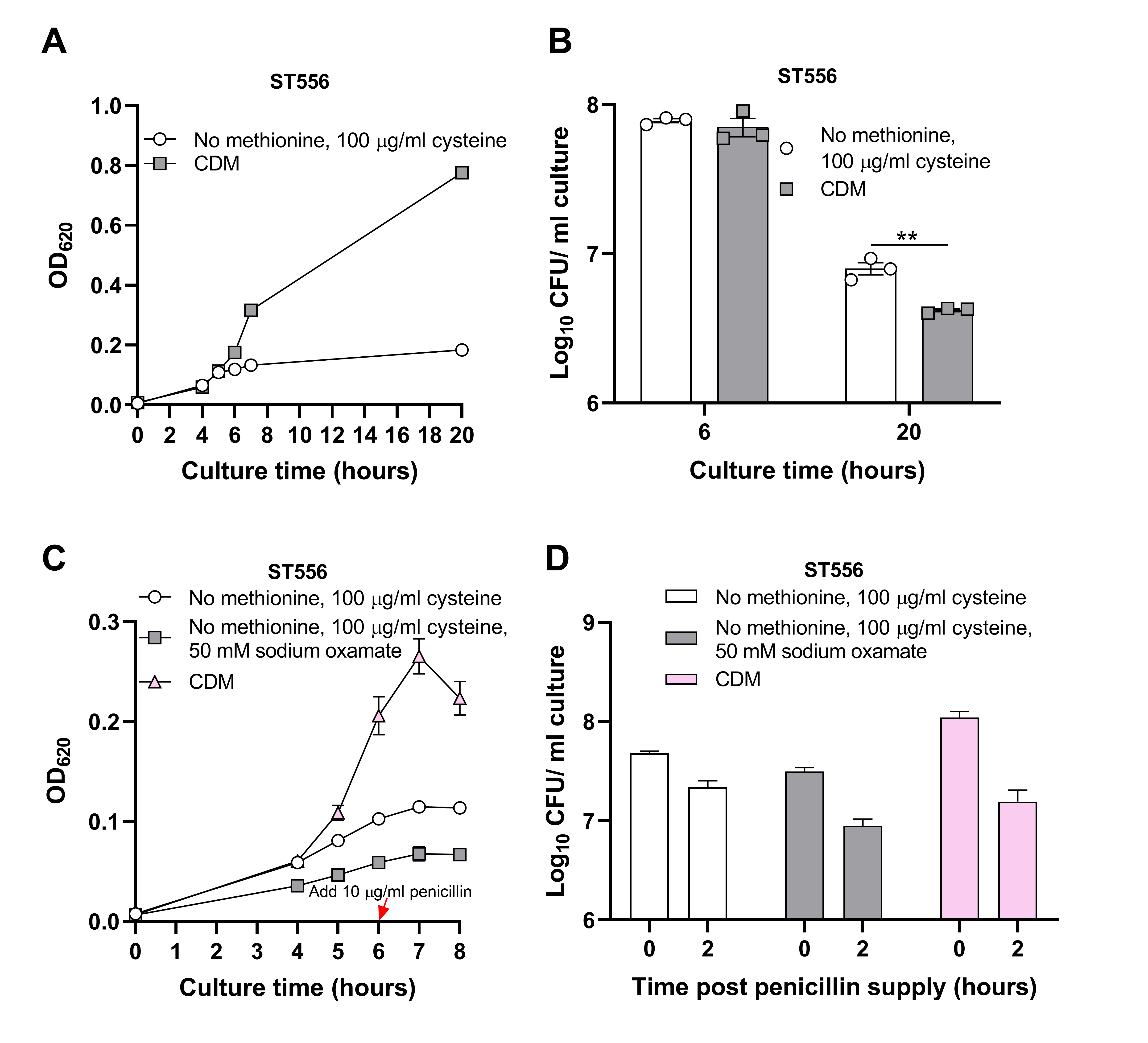

Supplement: S4 Fig — A, Growth curves (OD620) and B, Survival (CFU) of ST556 cultured in CDM with no methionine and 100 μg/ml cysteine or standard CDM. C, Growth curves (OD620) and D, Survival (CFU) of ST556 cultured in CDM with no methionine and 100 μg/ml cysteine, no methionine, 100 μg/ml cysteine and 50 mM sodium oxamate, or standard CDM. Penicillin was added at 6 hr post inoculation. At 0 and 2 hr post inoculation, bacterial CFU was determined. Each experiment was conducted in triplicate samples. P values < 0.01 (**). (TIF) [file ppat.1014381.s004.tif]
