# Supplementary material for: Eliminate pneumococcal colonization by targeting intracellular acidification that promotes H2O2 production to enhance bacterial survival
Source: PLoS Pathog. 2026 Jun 23;22(6):e1014381. doi: 10.1371/journal.ppat.1014381 (PMC13313337; doi:10.1371/journal.ppat.1014381)
Supplement: S1 Table — (DOCX) [file ppat.1014381.s005.docx]

**S1 Table. Bacterial strains used in this study**

**REFERENCES**

| **Strain** | **Description** | **Reference or Source** |
| --- | --- | --- |
| TH4306 | *Streptococcus pneumoniae* strain D39 derivative; *rpsL1* | ^1^ |
| TH9660 | TH4306Δ*metE* | ^2^ |
| LS3 | TH4306 derivative; plasmid pIB166 with pH-GFP was transformed into TH4306; TH4306 pIB166-pH-GFP | This study |
| LS7 | TH9660 derivative; plasmid pIB166 with pH-GFP was transformed into TH9660; TH9660 pIB166-pH-GFP | This study |
| ST556 | *Streptococcus pneumoniae* strain, serotype 19F, encapsulated | ^3^ |
| LS8 | ST556 derivative; plasmid pIB166 with pH-GFP was transformed into ST556; ST556 pIB166-pH-GFP | This study |
| LS9 | TH9660 derivative; the promoter of *ldh* was replaced by the promoter of SPD0818 in TH9660 background; TH9660 P*ldh*::P0818 | This study |
| LS10 | LS9 derivative; plasmid pIB166 with pH-GFP was transformed into LS9; LS9 pIB166-pH-GFP | This study |
| LS11 | TH4306 derivative; the promoter of *ldh* was replaced by the promoter of SPD0818 in TH4306 background; TH4306 P*ldh*::P0818 | This study |
| LS12 | LS11 derivative; plasmid pIB166 with pH-GFP was transformed into LS11; LS11 pIB166-pH-GFP | This study |
| LS13 | TH9660 derivative; *spxB* gene was removed in TH9660 background; TH9660Δ*spxB* | This study |
| LS14 | TH4306 derivative; *spxB* gene was removed in TH4306 background; TH4306Δ*spxB* | This study |
| LS15 | TH9660 derivative; *lctO* gene was removed in TH9660 background; TH9660Δ*lctO* | This study |
| LS16 | LS13 derivative; *lctO* gene was removed in LS13 background; LS13Δ*lctO* | This study |

1 Wen ZS, Sertil O, Cheng YX, Zhang SS, Liu X, Wang WC, et al. Sequence Elements Upstream of the Core Promoter Are Necessary for Full Transcription of the Capsule Gene Operon in *Streptococcus pneumoniae* Strain D39. Infect Immun. 2015;83:1957-1972.

2 Zhang C, An H, Hu J, Li J, Zhang W, Lan X, et al. MetR is a molecular adaptor for pneumococcal carriage in the healthy upper airway. Mol Microbiol. 2021; 116:438-458.

3 Li G, Hu FZ, Yang X, Cui Y, Yang J, Qu F, et al. Complete genome sequence of *Streptococcus pneumoniae* strain ST556, a multidrug-resistant isolate from an otitis media patient. J Bacteriol. 2012;194:3294-3295.
