# Supplementary material for: Eliminate pneumococcal colonization by targeting intracellular acidification that promotes H2O2 production to enhance bacterial survival
Source: PLoS Pathog. 2026 Jun 23;22(6):e1014381. doi: 10.1371/journal.ppat.1014381 (PMC13313337; doi:10.1371/journal.ppat.1014381)
Supplement: S2 Table — (DOCX) [file ppat.1014381.s006.docx]

**S2 Table. Primers used in this study**

| **Primer number** | **Sequence (5’-3’)** |
| --- | --- |
| Pr0029 | CCTTGGTAGTCACTTCAGGATTATC |
| Pr0030 | GAGATCTAGATTTTAAAAAACTTTGTCACGAATAT |
| Pr0031 | GAGACTCGAGATGACTTCAACTAAACAACACAAAA |
| Pr0032 | TCAAGGATTGCTTTAGTGATACGAG |
| Pr0037 | GGTCTCGCCTTTTAAAAAACTTTGTCACGAATAT |
| Pr0038 | GGTCTCGAAGGAAGTTAGCTTACTAAAAAAATGT |
| Pr0039 | GGTCTCGATCTTCTACCCCCAACTTCTTTATCTT |
| Pr0040 | GGTCTCGAGATGACTTCAACTAAACAACACAAAA |
| Pr0041 | ctgctaacccagttgacgttttgac |
| Pr0042 | CGTGCACTGAACGAGCATCCACATC |
| Pr0053 | gactacattgaagcccttgaatacg |
| Pr0054 | GAGATCTAGAAAACTGTCCTCCTTGATTAAGTAAG |
| Pr0055 | GAGACTCGAGtaaaacagattgcctccactgaatg |
| Pr0056 | AGAAGGCATCAAAGAAGTGATAATC |
| Pr0057 | GGTCTCGTAAAACTGTCCTCCTTGATTAAGTAAG |
| Pr0058 | GGTCTCGtttaaaacagattgcctccactgaatg |
| Pr7932 | GATTGCCATCATGAGTGACAAGG |
| Pr7933 | AGTGTCCACTTCGCGAAGGGT |
| Pr14761 | ccgaacagtcatgctgacaggagac |
| Pr14762 | GAGATCTAGACCCTTGAGTCATAATGATAACTCTC |
| Pr14763 | GAGACTCGAGgcaattaaataattcctctcgccga |
| Pr14764 | CCTTAAAGATTCCGACTAATATTGG |
| Pr14765 | GGTCTCGGCCCCTTGAGTCATAATGATAACTCTC |
| Pr14766 | GGTCTCGgggcaattaaataattcctctcgccga |
